# Metabolic Turnover of Synaptic Proteins: Kinetics, Interdependencies and Implications for Synaptic Maintenance

**DOI:** 10.1371/journal.pone.0063191

**Published:** 2013-05-02

**Authors:** Laurie D. Cohen, Rina Zuchman, Oksana Sorokina, Anke Müller, Daniela C. Dieterich, J. Douglas Armstrong, Tamar Ziv, Noam E. Ziv

**Affiliations:** 1 Technion Faculty of Medicine, Lorry Lokey Center for Life Sciences and Engineering, Technion, Haifa, Israel; 2 Network Biology Research Laboratories, Lorry Lokey Center for Life Sciences and Engineering, Technion, Haifa, Israel; 3 Smoler Proteomics Center, Faculty of Biology, Technion, Haifa, Israel; 4 Institute for Adaptive and Neural Computation, University of Edinburgh, Edinburgh, United Kingdom; 5 Leibniz-Institute for Neurobiology, Magdeburg, Germany; 6 Institute for Pharmacology and Toxicology, Otto-von-Guericke University, Magdeburg, Germany; University of Michigan, United States of America

## Abstract

Chemical synapses contain multitudes of proteins, which in common with all proteins, have finite lifetimes and therefore need to be continuously replaced. Given the huge numbers of synaptic connections typical neurons form, the demand to maintain the protein contents of these connections might be expected to place considerable metabolic demands on each neuron. Moreover, synaptic proteostasis might differ according to distance from global protein synthesis sites, the availability of distributed protein synthesis facilities, trafficking rates and synaptic protein dynamics. To date, the turnover kinetics of synaptic proteins have not been studied or analyzed systematically, and thus metabolic demands or the aforementioned relationships remain largely unknown. In the current study we used dynamic Stable Isotope Labeling with Amino acids in Cell culture (SILAC), mass spectrometry (MS), Fluorescent Non–Canonical Amino acid Tagging (FUNCAT), quantitative immunohistochemistry and bioinformatics to systematically measure the metabolic half-lives of hundreds of synaptic proteins, examine how these depend on their pre/postsynaptic affiliation or their association with particular molecular complexes, and assess the metabolic load of synaptic proteostasis. We found that nearly all synaptic proteins identified here exhibited half-lifetimes in the range of 2–5 days. Unexpectedly, metabolic turnover rates were not significantly different for presynaptic and postsynaptic proteins, or for proteins for which mRNAs are consistently found in dendrites. Some functionally or structurally related proteins exhibited very similar turnover rates, indicating that their biogenesis and degradation might be coupled, a possibility further supported by bioinformatics-based analyses. The relatively low turnover rates measured here (∼0.7% of synaptic protein content per hour) are in good agreement with imaging-based studies of synaptic protein trafficking, yet indicate that the metabolic load synaptic protein turnover places on individual neurons is very substantial.

## Introduction

Chemical synapses contain multitudes of proteins, some of which play direct roles in synaptic transmission, whereas others regulate synaptic function or serve as structural scaffolds. Proteins, including synaptic ones, have finite lifetimes and therefore, need to be continuously replaced with freshly synthesized copies. Given the huge numbers of synaptic connections each central nervous system neuron makes, maintenance of synaptic contents would conceivably place enormous metabolic demands on individual neurons. These demands in turn, depend on anabolic and catabolic rates of synaptic proteins. Surprisingly, perhaps, the turnover kinetics of synaptic proteins have not yet been studied systematically. As a result, the estimates for such kinetics vary widely. Whereas older studies based on radiolabeling methods indicated that the half-lives of some presynaptic proteins can be remarkably long (e.g. [1], [2]), more recent *in vitro* studies have reported half-lives of synaptic proteins in the range of several hours (e.g. [3], [4]). Thus, the metabolic cost of maintaining synapses remains largely unknown.

The elaborate, anisotropic architecture of neurons poses unique challenges in terms of synaptic proteostasis: First, synapses, and in particular presynaptic compartments, are often located at enormous distances from the major site of protein synthesis, namely the neuronal cell body. Given the enormous lengths axons can attain, it might be expected that the life-spans of presynaptic proteins would generally be longer than those belonging to somatodendritic compartments. Neurons, however, contain sophisticated and quite efficient transport mechanisms for delivering particular proteins to the far reaches of axons. Yet the transport rates of other synaptic proteins can be rather slow – on the order of a few millimeters per day [5]–[8]. In addition, substantial evidence has accumulated for local synthesis of synaptic proteins in dendrites (reviewed in [9]–[12]) and possibly in axons [13], [14]. Therefore, relationships between turnover rates of particular synaptic proteins and their cellular localization are currently unknown. Moreover, despite much evidence for local protein synthesis in dendrites and axons, it is generally thought that most synaptic proteins, and in particular presynaptic proteins, are transported from the cell body (e.g. [15]; but see [16]). It thus remains unclear how the short lifetimes reported for some synaptic proteins (e.g. [3], [4]) are compatible with the relatively long times required for trafficking them to their remote destinations (reviewed in [16]).

Beyond continual replenishment, protein synthesis is believed to play essential roles in driving long-term changes in synaptic composition and function. Moreover, local synthesis and degradation processes have been suggested to affect the properties of specific synapses by changing the abundance of particular synaptic molecules in a spatially confined manner (reviewed in [12], [17]). On the other hand, numerous live imaging studies suggest that synaptic molecules – receptors, scaffolding, cytoskeletal and signaling molecules alike – continuously move in, out and between synapses at fairly rapid rates (reviewed in [18]–[24]). Such continuous interchange would seem to defeat the purported specificity of local synthesis, unless metabolic turnover rates are roughly equivalent to such interchange rates. At present, however, as metabolic turnover rates of synaptic proteins have not been systematically studied, resolving functional relationships between synaptic protein interchange, protein synthesis and synaptic plasticity in a manner that is constrained by physiological evidence is not possible.

In the current study we set out to systematically measure and analyze the metabolic half-lives of synaptic proteins, assess the metabolic load imposed on neurons by the need to continuously synthesize synaptic proteins, examine potential relationships between synaptic protein turnover rates, cellular localization and association with particular molecular complexes, and compare the metabolic turnover rates of specific synaptic proteins with the exchange rates of those molecules. The findings and their implications are described next.

## Results

### Metabolic Turnover Rates of Synaptic Proteins Measured by Dynamic SILAC and MS

To measure metabolic turnover rates of synaptic proteins we used dynamic SILAC (Stable Isotope Labeling with Amino acids in Cell culture) and mass spectrometry (MS) [25]–[31]. This approach is based on the replacement of select amino acids (AAs) in growth media with similar AAs containing non-radioactive heavy isotopes of particular atoms. With time, these labeled (“heavy”) AAs are incorporated into newly synthesized proteins, whereas the degradation of preexisting proteins is associated with the gradual loss of proteins containing “light” (i.e. unlabeled) versions of these AAs. At particular time points, cells are lysed, and protein extracts are digested into short peptides, which are thereafter subjected to MS analysis. For each peptide analyzed and identified, a ratio of heavy to light peptide abundance is calculated, providing a fractional measure of newly synthesized copies for that particular protein species. By repeating this process at several time points, metabolic turnover rates for thousands of proteins can be measured (e.g. [32]).

**Figure 1:**
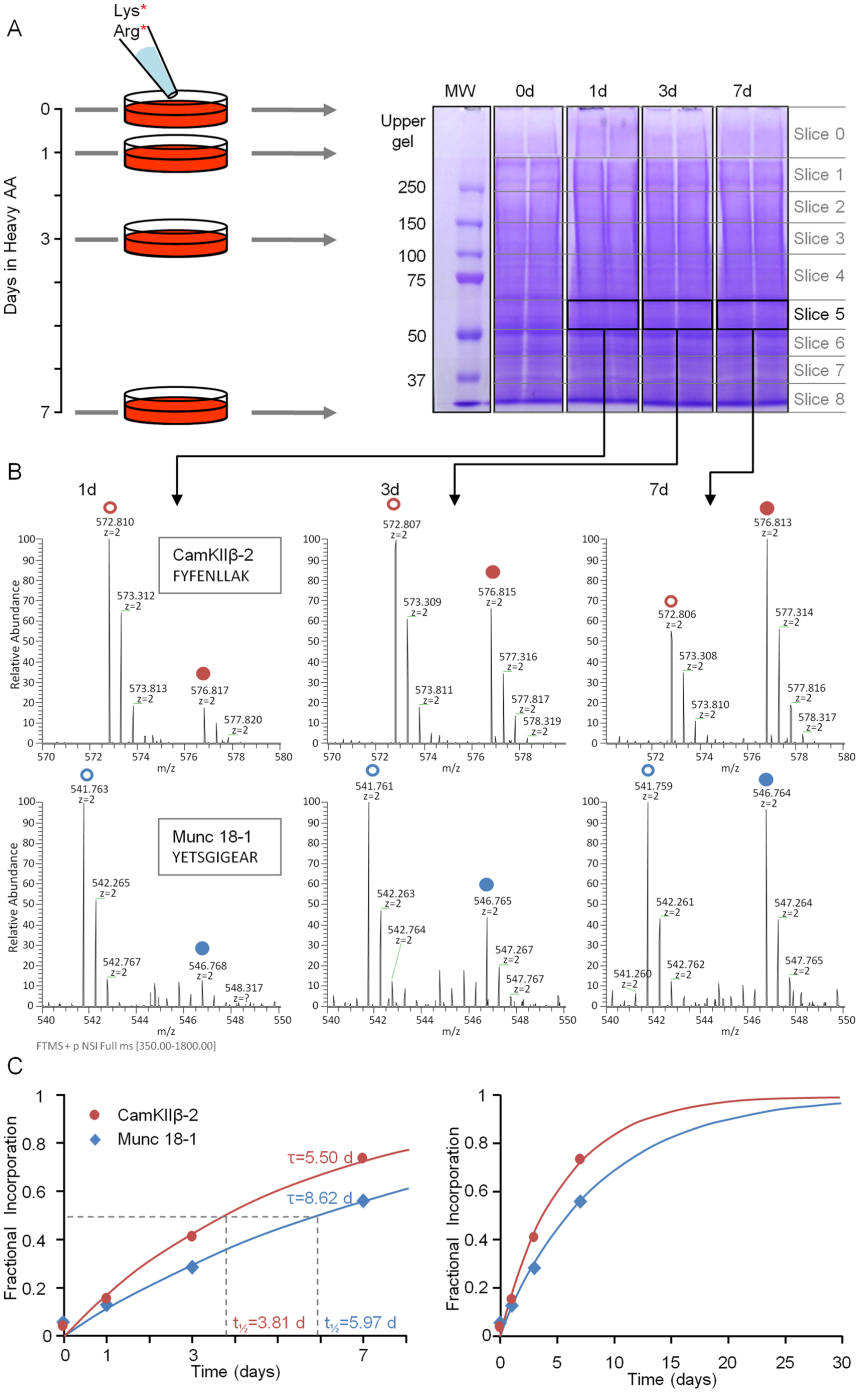
Measuring metabolic protein turnover by SILAC and MS. **A)** Illustration of the experimental process. At t = 0, heavy lysine and arginine were added to the media of cortical neurons in primary culture (14 days *in vitro*). 0, 1, 3 and 7 days afterward, cells were harvested and separated side by side by SDS-PAGE. One such gel (stained with Coomassie Blue) is shown on right. Two lanes were run for each time-point to increase protein amounts. Gels were then cut into 9 slices as indicated, proteins in each slice were digested, and the resulting peptides from each slice and each time point were submitted separately to MS analysis. **B)** MS spectrogram showing the relative amounts, at three time points, of light (open circles) and heavy (closed circles) populations of two particular peptides from slice 5. **C)** Heavy AA incorporation rates for two particular proteins (Munc18-1 and CaMKII-β2). Each data point represents the fractional incorporation values averaged for all peptides belonging to these particular proteins at a given time point. All four data points were used for fitting to exponential curves (solid lines), providing estimates of time constants (τ) and half-lives as indicated. Graph on right hand side shows extrapolation of same exponential curves to longer times.

All experiments were carried out in rat cortical neurons, raised in culture for two weeks. Typically, dynamic SILAC experiments require abrupt and complete media exchanges to assure a full substitution of light AAs with their heavy counterparts. In neuronal cell cultures, during the stage at which most synaptogenesis has been completed (2–3 weeks in culture), aggressive washes and complete media exchanges are severely detrimental to neuronal viability. Therefore, rather than replace media, we added an excess of heavy lysine and arginine. Specifically, after 14 days in culture, heavy lysine and arginine were added to the media, resulting in final concentrations of ∼1.9 and ∼2.9 mM, respectively, and final heavy to light (H/L) ratios of ∼5∶1 for both lysine and arginine. 0, 1, 3, or 7 days later, the neurons were lysed and extracted; the extracts were separated on polyacrylamide gels, which were subsequently cut into 9 sets of bands according to molecular weight. Each gel slice was then subjected to MS analysis, and an H/L ratio for each identified peptide was determined. H/L ratios for all peptides belonging to a particular protein species were pooled, providing an average H/L ratio for each protein. The entire process is illustrated in Fig. 1.

The procedure described above involved exposure to elevated levels of lysine and arginine. 6x lysine and arginine (heavy or light) concentrations, however, did not noticeably affect neuronal viability, nor did they reduce synaptic densities as assayed by immunolabeling against the postsynaptic density protein PSD-95 (data not shown). Furthermore, profiles of MS-based protein identifications were nearly identical to those obtained in control preparations (Fig. S1). Finally, no effects on spontaneous activity levels were observed when network activity was quantified by multielectrode array recordings after the addition of heavy lysine and arginine as described above (Fig. S2). Collectively these data indicate that elevated lysine and arginine concentrations did not significantly affect viability, activity or neuronal properties.

**Figure 2:**
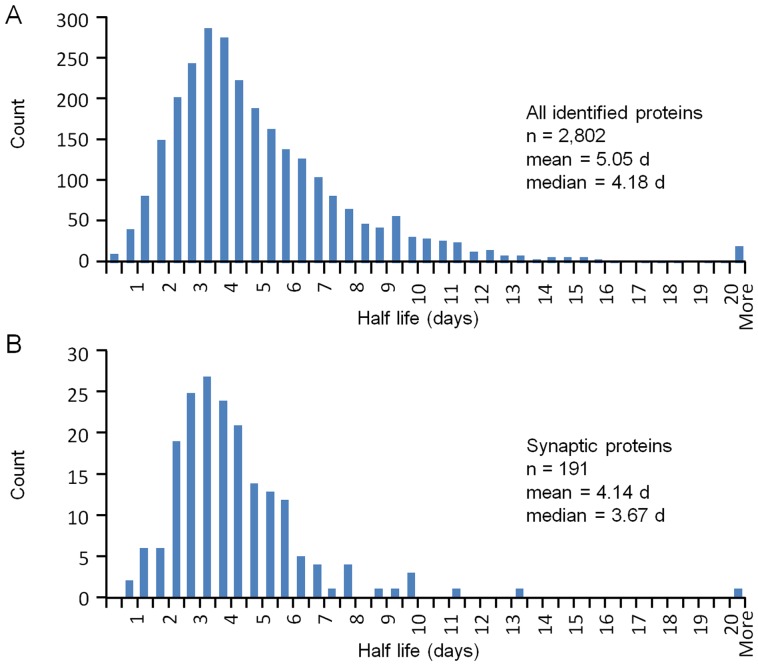
Distributions of metabolic half-life estimates. **A)** Distribution of metabolic half-life estimates for all identified proteins for which fractional incorporation data was obtained for all four time points. Proteins for which fits to single exponentials were not satisfactory (∼2%) were excluded. **B)** Distribution of metabolic half-life estimates for 191 synaptic proteins (Table 1).

Altogether 6 separate experiments were performed (two full, four time point experiments and four single time point experiments). Data were pooled as described in Materials and Methods and subsequently analyzed under the following assumptions: 1) the total amount (H+L) of each protein species was constant over time (but see below), and therefore, incorporation rates of heavy AAs, which reflect protein synthesis, are balanced by the loss rates of light AAs, which reflect protein degradation; 2) heavy AA incorporation and light AA loss are expected to follow single exponential kinetics; 3) the maximal H/L ratio expected is the H/L ratio for lysine and arginine in the growth medium (5∶1, in these experiments). H/L ratios for all time points were converted into fractional incorporation ratios ranging from 0 (no incorporation of heavy AAs) to 1.0 (full replacement of light AAs with heavy AAs), after correcting to the maximal possible ratio (∼0.828; third assumption mentioned above). The corrected fractional ratios at all four time points were fit to single exponential curves and finally, the resulting time constants of these fits were converted to the more commonly used half-life (*t_½_*) measures (see Materials and Methods for further details). This process is exemplified for the synaptic proteins Munc18-1 and CaMKIIβ-2 in Fig. 1B,C. Altogether we identified 4,438 proteins. Out of these, data were obtained at 4 time points for 2,859 identified proteins, including tens to hundreds of synaptic proteins (depending on the definition of a synaptic protein). Fits to single exponential curves were good to excellent for >92% of identified proteins (Fig. S3). Proteins for which fits were unacceptable (∼2%) were not examined further, resulting in satisfactory half-life estimates for 2,802 proteins (Fig. 2; Table S1).

**Figure 3:**
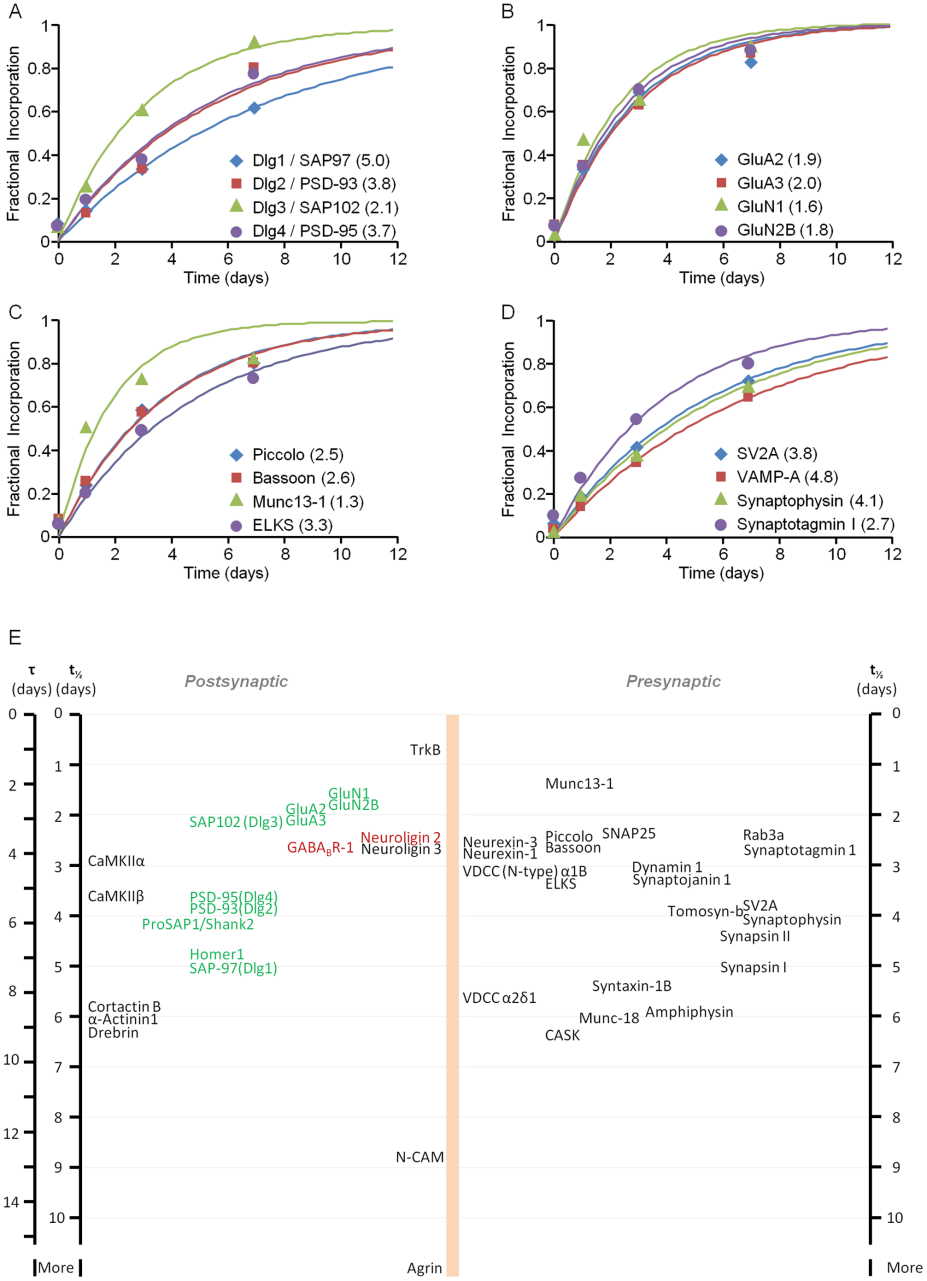
Metabolic half-life estimates for well characterized synaptic proteins. **A–D)** Heavy AA incorporation rates for four groups of synaptic proteins**:** Glutamatergic synapse Dlg family of scaffolding molecules (**A**); glutamate receptor subunits (**B**); cytoskeleton of the active zone (CAZ) molecules (**C**); and synaptic vesicle molecules (**D**). Each data point (as in Fig. 1C) represents heavy AA fractional incorporation values averaged for all peptides belonging to that particular protein at a given time point. The solid lines represent best fits to single exponential curves. Half-life estimates (in days) based on these fits are provided in the legend (brackets). **E)** Metabolic half-life estimates for a select group of synaptic proteins. Proteins associated primarily with glutamatergic and GABAergic synapses are shown in green and red respectively. Note that proteins with very similar half-lives were sometimes separated slightly to increase readability.

The vast majority of identified proteins exhibited relatively slow turnover rates (mean: 5.05 days, median: 4.18 days), with half-lives ranging from 5 hours or less to more than 50 days (Fig. 2A). To evaluate the half-lives of synaptic proteins, we collated a list of 191 proteins that are either synapse-specific, highly enriched in synaptic compartments, or implicated in synaptic function (synaptic vesicle proteins, proteins involved in synaptic vesicle recycling, active zone proteins, neurotransmitter receptors, postsynaptic scaffolding molecules, adhesion molecules implicated in synaptic organization, and others; Table 1). As shown in Fig. 2B, these were also quite broadly distributed, although to a somewhat lesser extent. Here too, relatively slow turnover rates were observed (mean: 4.14 days, median: 3.67 days) ranging from 17 hours (TrkB) to 23 days (Agrin). Examples for select groups of synaptic proteins are shown in Fig. 3A–D, and schematically in Fig. 3E. Although the half-life estimates described above were based on data pooled from all experiments, half-life estimates based on single experiments correlated extremely well with each other (*r* = 0.924; Fig. S4).

**Table 1:** Synaptic and synaptically related proteins.

Gene Symbol	Common name	t _½_ (days)	Gene Symbol	Common name	t _½_ (days)	Gene Symbol	Common name	t _½_ (days)
Ntrk2	TrkB isoform 1	0.72	Rab4a	Rab-4A	3.08	Syn2	Synapsin II	4.40
Nedd4	NEDD4	0.78	Cacna1b	Cav2.2	3.09	Dbnl	Drebrin-like	4.43
Atxn10	Ataxin 10	1.08	Rab3gap2	Rab3 GTPase	3.10	Syngr3	Synaptogyrin 3	4.55
Ngef	Ephexin-1	1.15	Fmr1	FMR1	3.11	Sh3gl2	Endophilin A1	4.59
Cntnap1	Caspr1/Neurexin-4	1.18	Eef2	Elongation factor 2	3.11	Mtor	mTOR	4.61
Unc13a	Munc-13-1	1.32	Dnm1l	Dynamin 1-like	3.14	Bcan	Brevican core 1	4.63
Ncdn	Norbin	1.39	Cadps	CAPS-1	3.15	Actn4	Alpha-actinin-4	4.67
Nes	Nestin	1.43	Arap1	Centaurin δ2 (ArfGAP 1)	3.16	Eef1a1	Elongation factor 1-alpha 1	4.69
Mpp5	MAGUK p55 subfamily 5	1.51	Ppfia2	Liprin α2	3.18	Canx	Calnexin	4.78
Grin1	NMDAR1/NR1	1.61	Dclk2	DCLK2	3.19	Vapa	VAMP-A	4.79
Grin2b	NMDAR2B/NR2B	1.80	Synj1	Synaptojanin 1	3.25	Homer1	Homer homolog 1	4.82
Ptprs	LAR-PTP2	1.91	Ppfia3	Liprin α3	3.27	Snap91	SNAP-91	4.86
Epha4	Eph receptor A4	1.95	Rab8a	Rab-8A	3.27	Syn1	Synapsin I	4.95
Gria2	GluA2	1.95	Scamp1	Scamp1	3.29	Stxbp3	Syntaxin binding 3	4.96
Gria3	GluA3	2.04	Hip1r	Huntingtin interacting 1	3.29	Ap2a2	AP-2 α2	4.98
Ppp1r9a	Neurabin 1	2.08	Erc1	ELKS/Erc1/CAST2	3.30	Agap3	Centaurin γ3 (ArfGAP3)	4.99
Stx6	Syntaxin 6	2.10	Rab5c	Rab 5c	3.32	Dlg1	Dlg1/SAP-97	5.01
Dlg3	Dlg3/SAP-102	2.13	Sv2b	SV2B	3.36	Camk2g	CaMKIIγ	5.03
Syne1	Nesprin 1	2.15	Map2	MAP2	3.36	Vapb	VAMP-B	5.10
Fxr2	Fxr2	2.18	Macf1	Liprin β1	3.45	Ap2a2	AP-2 α2	5.17
Rab3a	Rab 3a	2.26	Scfd1	Syntaxin-binding 1-like	3.46	Cd47	CD47	5.20
Rasa3	RasGAP3	2.27	Rab5b	Rab 5b	3.52	Rptor	Raptor	5.21
Lphn1	Latrophilin-1	2.29	Cyfip2	FMR1 interacting 2	3.55	Syngr1	Synaptogyrin 1	5.22
Cdh2	N-cadherin	2.31	Rph3a	Rabphilin 3A	3.55	Asap2	ArfGAP2	5.23
Snap25	SNAP-25	2.31	Napg	NSF attachment γ soluble	3.56	Lin7c	lin-7 homolog C	5.29
Camk4	CaMKIV	2.37	Ptpra	PTPRA	3.59	Ap2m1	AP-2 µ	5.35
Ppfia4	Liprin α4	2.40	Cdk5	CDK5	3.59	Sept3	Septin 3	5.36
Epha5	Eph receptor A5	2.41	Nsf	NSF	3.59	Negr1	Kilon	5.38
Kctd12	KCTD12	2.43	Srcin1	SNAP-25 interacting (SNIP)	3.60	Stx1b	Syntaxin 1B	5.40
Rab3a	Rab-3A	2.45	Atp6v0a1	SV proton pump 116 kDa	3.63	Plxnd1	Plexin-D1	5.60
Snap29	SNAP-29	2.45	Atp6v1a	ATPase, H+ subunit A	3.65	Cacna2d1	Cacna2d1	5.60
Rab11b	Rab 11b	2.46	Dlg4	Dlg4/PSD-95	3.67	Caskin1	Caskin-1	5.66
Napa	SNAP-α	2.48	Rab5a	Rab 5a	3.68	Ctnna2	Catenin α2	5.71
Camk1d	CaMKIδ	2.50	Naa15	NMDAR regulated 1	3.69	L1cam	Ncam L1	5.73
Pclo	Piccolo	2.54	Ap3d1	AP-3 δ1	3.70	Ap2a1	AP-2 α1	5.90
Nlgn2	Neuroligin 2	2.56	Htt	Huntingtin	3.75	Amph	Amphiphysin	5.93
Bsn	Bassoon	2.57	Pacsin1	Syndapin 1	3.77	Ap2b1	AP-2 β	5.95
Git2	ArfGAP2 GIT2	2.57	Dlg2	Dlg2/PSD-93	3.80	Stxbp1	Munc18-1	5.98
Nrcam	NrCAM	2.58	Sv2a	SV2a	3.80	Itsn1	Intersectin 1	5.98
Rab7a	Rab 7a	2.60	Camk2b	CaMKII β2	3.81	Cttn	Cortactin isoform B	5.98
Nlgn3	Neuroligin 3	2.61	Mllt4	Afadin	3.82	Sept5	Septin 5	5.99
Nrxn3	Neurexin 3α	2.61	Stx7	Syntaxin 7	3.83	Adap1	Centaurin α1 (ArfGAP1)	6.01
Rufy3	Singar 2	2.64	Dnm1l	Dynamin 1-like	3.83	Actn1	Brain-specific α actinin	6.06
Gabbr1	GABA-BR1	2.70	Stxbp5	Tomosyn	3.91	Dbn1	Drebrin	6.27
Syt1	Synaptotagmin 1	2.74	Dtnb	Dystrobrevin β	3.96	Nptn	Neuroplastin	6.32
Ctnnb1	Catenin β1	2.78	Camkv	CaMKV	4.02	Cask	CASK	6.35
Mpp2	MAGUK p55 subfamily 2	2.78	Cyfip1	FMR1 interacting 1	4.04	D10Wsu52e	SynGAP1 homolog	6.56
Hip1	Huntingtin-interacting 1	2.79	Sirpa	Sirpa	4.05	Vat1	VAT-1 homolog	6.60
Syngap1	SynGAP1	2.82	Akap5	Akap5	4.06	Adap1	Centaurin α (ArfGAP1)	6.93
Vamp7	VAMP-7	2.86	Kcnab2	Kv-β2	4.09	Nfasc	Neurofascin	6.97
Nrxn1	Neurexin 1	2.89	Syp	Synaptophysin	4.09	Epb41l1	Band 4.1-like	7.46
Kalrn	Kalirin	2.91	Abi1	Abelson interactor 1	4.09	Tln2	Talin 2 isoform 3	7.51
Napb	NSF attachment β soluble	2.92	Cttnbp2	Cortactin-binding protein 2	4.10	Ppm1f	CaM kinase phosphatase	7.61
Syngr2	Synaptogyrin 2	2.92	Epn1	Epsin 1	4.13	Sntb1	Syntrophin β1	7.62
Slc12a5	Neuronal K-Cl cotransporter	2.92	Arl6ip5	PRA1 family 3	4.14	Stx4	Syntaxin 4A	7.91
Hip1r	Huntingtin interacting 1-rltd	2.95	Shank2	Shank2/ProSAP1	4.14	Ncam1	N-Cam1	8.80
Acap2	Centaurin β2 (ArfGAP 2)	2.97	Sh3gl1	Endophilin A2	4.18	Palm	Paralemmin-1	9.41
Rtn4	Nogo	2.97	Plxna3	Plexin-A3	4.19	Sept11	Septin 11	9.58
Dnm1	Dynamin 1	3.02	Dclk1	DCLK1	4.20	Marcksl1	MARCKS-related	9.64
Camk2a	CaMKIIα	3.02	Camk2d	CaMKIIδ	4.21	Tnr	Tenascin R	9.99
Tecr	Synaptic glycoprotein SC2	3.03	Syncrip	Synaptotagmin-binding	4.23	Sypl1	Synaptophysin-like 1	11.46
Ppp1r9b	Neurabin 2	3.04	Stx16	Syntaxin 16	4.26	Tnc	Tenascin C	13.04
Nedd4l	NEDD4-like	3.06	Bcan	Brevican core	4.28	Agrn	Agrin	23.59
Rab3c	Rab 3c	3.07	Dnm2	Dynamin 2	4.36			

List of 191 synaptic and synaptically related proteins and their respective metabolic half-life estimates (in days). The maximal accepted SSE value for this data set was 0.08.

As mentioned above, a key assumption in these experiments was that the total amount (H+L) of each protein species remained constant and thus, incorporation rates represented the complement of degradation rates. Synapse numbers, however, increase at moderate rates during the one week period used here. To quantify changes in synaptic numbers over these periods, we grew cortical neurons on thin-glass dishes under exactly the same experimental conditions and stained these preparations against the postsynaptic density protein PSD-95 at all four time points. It should be stressed that the cell cultures used here and elsewhere [33]–[34] are much denser than the sparse cell culture preparations typically used for cellular imaging experiments, for example, and are characterized by a very high density of synaptic connections, that is similar in many respects to the synaptic density observed in intact preparations (Fig. S5A). The synaptic density was quantified at 2 separate Z sections at all time points, resulting in a temporal profile of synaptic density over time (Fig. S5B). We observed that synaptic density increased by approximately 27% over one week (two separate experiments, 14 to 17 fields of view per time point per experiment). As exemplified in Figs. S5C,D, this increase in synaptic protein content over time would be expected to result in a slight *underestimate* of turnover rates. Interestingly, the fractional intensities of synaptic protein peptides within the total peptide mixture analyzed by MS barely changed over this period (Fig. S6).

In primary cultures of rat neurons, the period of two to three weeks *in vitro* represents the end of the rapid synaptogenesis phase and a transition into more mature states. To determine if turnover rates are slower in more mature preparations, we compared the fractional incorporation ratios for all identified proteins 3 days after exposure to heavy lysine and arginine in neurons exposed to these heavy AAs after two and three weeks in culture. Plotting the fractional incorporation ratios against each other (Fig. S7) revealed no trend, with a regression line exhibiting a slope of 1.006 (*r* = 0.92). Thus, no significant differences in overall protein metabolic turnover rates were observed between neurons maintained in culture for two or three weeks. Taken together, these findings suggest that a breach of the assumption of constant protein content was not a major confounding factor.

Finally, we repeated the dynamic SILAC experiment in a more conventional manner – that is, we grew cortical preparations in arginine and lysine free media to which labeled (“heavy”) variants of these AAs were added at nominal concentrations. After two weeks, the preparations were chased with media containing the unlabeled (“light”) isotopic forms of these AAs (see Materials and Methods). As before, neurons were lysed and extracted after 0, 1, 3, or 7 days and the digested extracts were subjected to MS analysis. The half-lives measured in this fashion correlated quite well with the estimates described above (*r* = 0.85, 1100 proteins identified in both data sets), but were 20–30% shorter (mean = 4.00 days; median = 3.42 days; 1501 proteins; Fig. S8). To exclude the possibility that these differences resulted from preparation to preparation variability, we performed in sister cell culture preparations a single time-point (3 days) SILAC experiment to compare the two protocols used here as well as the effects of media exchanges (washes) alone. Here too we noted that the half-life estimates for synaptic proteins obtained by “conventional” SILAC were slightly shorter (∼20%). We noted, however, that in “conventional” SILAC experiments, labeling with heavy AAs was not complete even after two weeks (reaching 85% ±6%), requiring some correction to the fractional incorporation values at the beginning of the chase period. Moreover, the washes involved in the chase procedure were also associated with some shortening of the half-life estimates (5–10%). Given these complications, we feel the latter estimates were less reliable, and therefore, all further analysis was limited to the data obtained in the experiments described in figures 1, 2, 3.

### Metabolic Turnover Rates of Synaptic Proteins Measured by FUNCAT and Quantitative Immunohistochemistry

The relatively slow turnover rates of synaptic proteins might seem surprising, given prior studies reporting synaptic proteins half-lives on the order of 4–20 hours in similar preparations [3]. It remained possible, however, that metabolic turnover rates reported here for whole cell extracts differ from metabolic turnover rates of synaptic proteins confined to synapses. We thus probed protein turnover rates at synapses using two different approaches.

**Figure 4:**
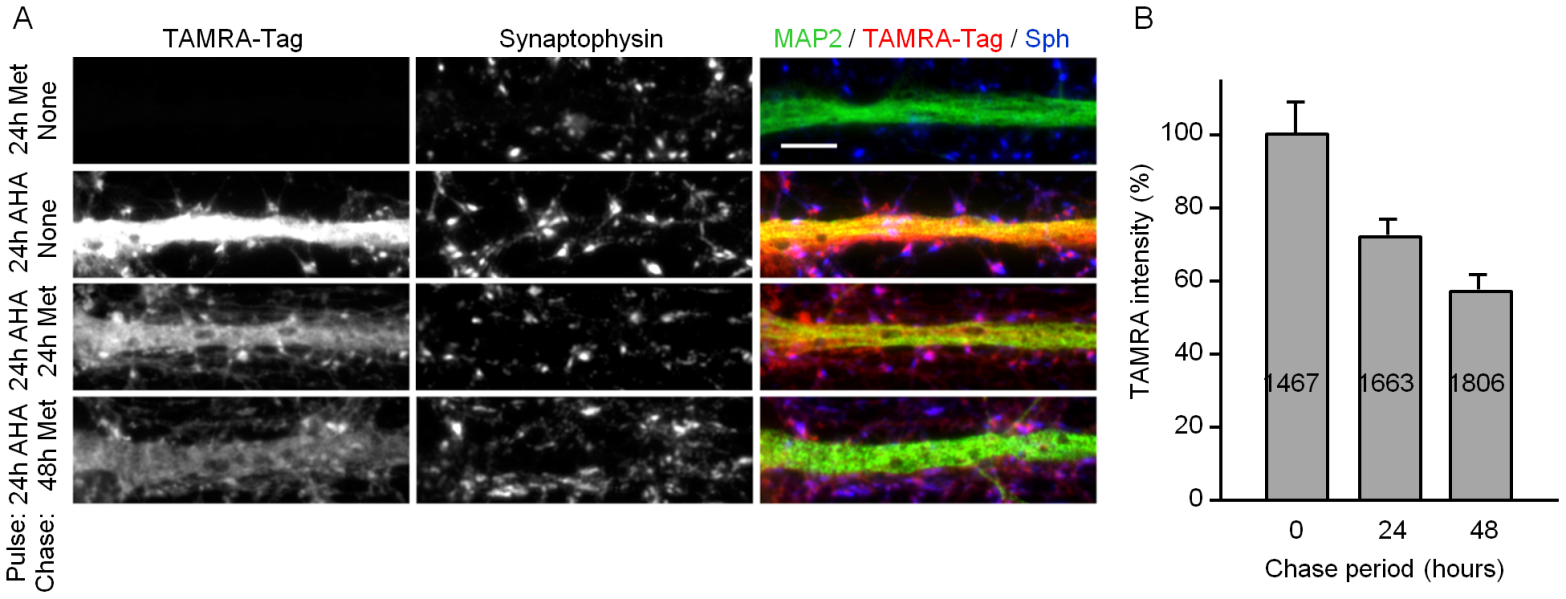
Degradation rates of newly synthesized proteins measured in dendritic spines. A 24 h pulse with 4 mM AHA was used to label newly synthesized proteins. Cells were subsequently fixed - immediately or after 24 or 48 h chase periods with high concentrations of methionine. Newly synthesized proteins (proteins containing AHA) were then visualized with a TAMRA-TAG using FUNCAT. **A)** Examples of proximal dendritic segments after visualization of newly synthesized proteins by FUNCAT, and after immunostaining against MAP2 and Synaptophysin (Sph). Note the strong TAMRA fluorescence in dendrites as well as in Synaptophysin positive synapses, and the reduction in TAMRA fluorescence after 24 and 48 h chase periods. Note also that no TAMRA fluorescence is observed in neurons that were not exposed to the AHA pulse (top row). Color coding: MAP2 - green, TAMRA-tag - magenta/red, Sph - blue. Scale bar: 5 µm. **B)** Quantification of TAMRA fluorescence intensity in synaptophysin-positive synapses following increasingly longer chase periods. Data is shown as average ± SEM. Data obtained from two independent experiments (two to three coverslips per experiment) and a total number of 40–46 proximal dendrites. The number of spines for which TAMRA-intensity was quantified is indicated inside the bars.

In the first, we employed FUNCAT (FlUorescent Non–Canonical Amino acid Tagging [35]) to visualize newly synthesized proteins and assess their degradation rates with a special focus on labeled proteins at synaptic compartments. A 24 h pulse with the non-canonical amino acid azidohomoalanine (AHA) allowed robust labeling of newly synthesized proteins in primary cultures of rat hippocampal neurons and subsequent visualization with a fluorescent Tetramethylrhodamine (TAMRA) tag using “click chemistry”. Abundant AHA-bearing proteins were detected within dendrites as well as in Synaptophysin positive synapses after a 24 h pulse (Fig. 4A). To measure global degradation rates of these newly synthesized proteins, preparations were chased for 24 h and 48 h with high concentrations of the natural amino acid methionine (4 mM) to stop the incorporation of AHA into nascent proteins [35]. We found that the fluorescence of pulse-labeled AHA-bearing proteins in synapses was reduced to 70% and 55% after 24 and 48 h respectively, as compared to samples fixed directly after 24 h pulse labeling (Fig. 4B).

**Figure 5:**
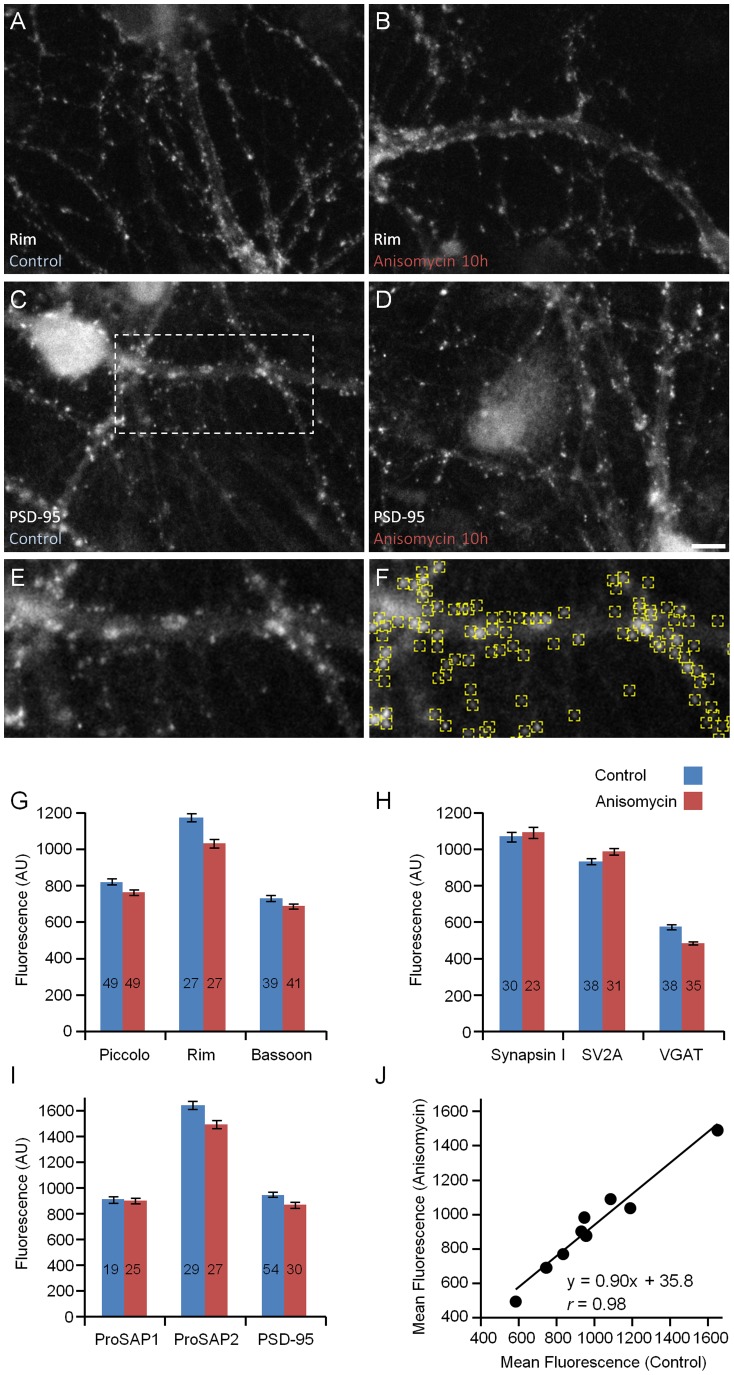
Minor loss of synaptic proteins from synaptic sites following suppression of protein synthesis for 10 hours. Quantitative immunocytochemistry of neurons exposed to the protein synthesis blocker anisomycin (25 µM) for 10 hours and thereafter labeled against nine different synaptic proteins. Neurons labeled against the CAZ protein Rim after exposure to carrier solution (**A**) or anisomycin (**B**). Neurons labeled against the PSD protein PSD-95 after exposure to carrier solution (**C**) or anisomycin (**D**). Scale bar, 10 µm. **E)** Enlarged view of region enclosed in the rectangle in C illustrating a programmatic localization of fluorescent puncta (**F**). Note that puncta are detected correctly regardless of their brightness. **G–I)** Changes in synaptic immuofluorescence levels measured following exposure to anisomycin for 10 hours (average ±SEM). Numbers within bars indicate the number of fields of view analyzed for each data set. Each field of view contained ∼297±122 puncta (average ± standard deviation). **J)** Average immuofluorescence levels following anisomycin treatment plotted against immunofluorescence levels in untreated neurons (same data as in panels G–I).

In a second approach, we exposed neurons maintained in culture for 14 days to the protein synthesis inhibitor anisomycin (25 µM) for 10 hours and used quantitative immunofluorescence directed against 9 synaptic proteins (Bassoon, Piccolo, Rim, Synapsin-I, VGAT, SV2A, PSD-95, ProSAP1/Shank2, ProSAP2/Shank3) to compare immunofluorescence levels in these preparations to those observed in matched preparations exposed only to carrier solution for the same time period. We found that synaptic immunofluorescence levels were only slightly reduced following 10 hours of exposure to anisomycin (Fig. 5A–I). In fact, plotting immunofluorescence levels of anisomycin treated neurons against control values for all 9 proteins indicated a general loss of <10% over this 10 hour period (Fig. 5J). Reductions in somatic immunofluorescence were similarly modest (data not shown). As a positive control we verified that anisomycin suppressed the expression of nuclear c-fos following exposure to low levels of glutamate (data not shown). These experiments are thus in good agreement with our SILAC analysis and further indicate that the metabolic half-lives of synaptic proteins are typically on the order of several days.

### Metabolic Turnover Rates, Cellular Compartmentalization and Functional Relationships

A comparison of metabolic turnover rates measured for synaptic proteins to those of the entire population of identified proteins indicates that the mean half-life measured for synaptic proteins is shorter than that measured for the entire population (4.17±0.17 vs. 5.06±0.07, mean±SEM, synaptic and entire population, respectively, p<10^−3^, Kolmogorov-Smirnov test). Comparison of half-life distributions (Fig. 2) indicates that the difference is mainly due to a long tail of proteins with slow turnover rates found in the general population. Gene Ontology (GO) based analysis performed using “Perseus” (http://www.maxquant.org/) and “GORILLA” (Gene Ontology enRIchment anaLysis and visuaLizAtion tool [36]), indicated that this long tail of relatively stable proteins is highly enriched in mitochondrial and extracellular matrix proteins (Figs. S9, S10A). Conversely, GO analysis indicated enrichment of Golgi apparatus-related proteins in the list of proteins with short half-lives, and to a lesser degree, protein degradation systems and dendritic shaft proteins (Fig. S10B).

**Figure 6:**
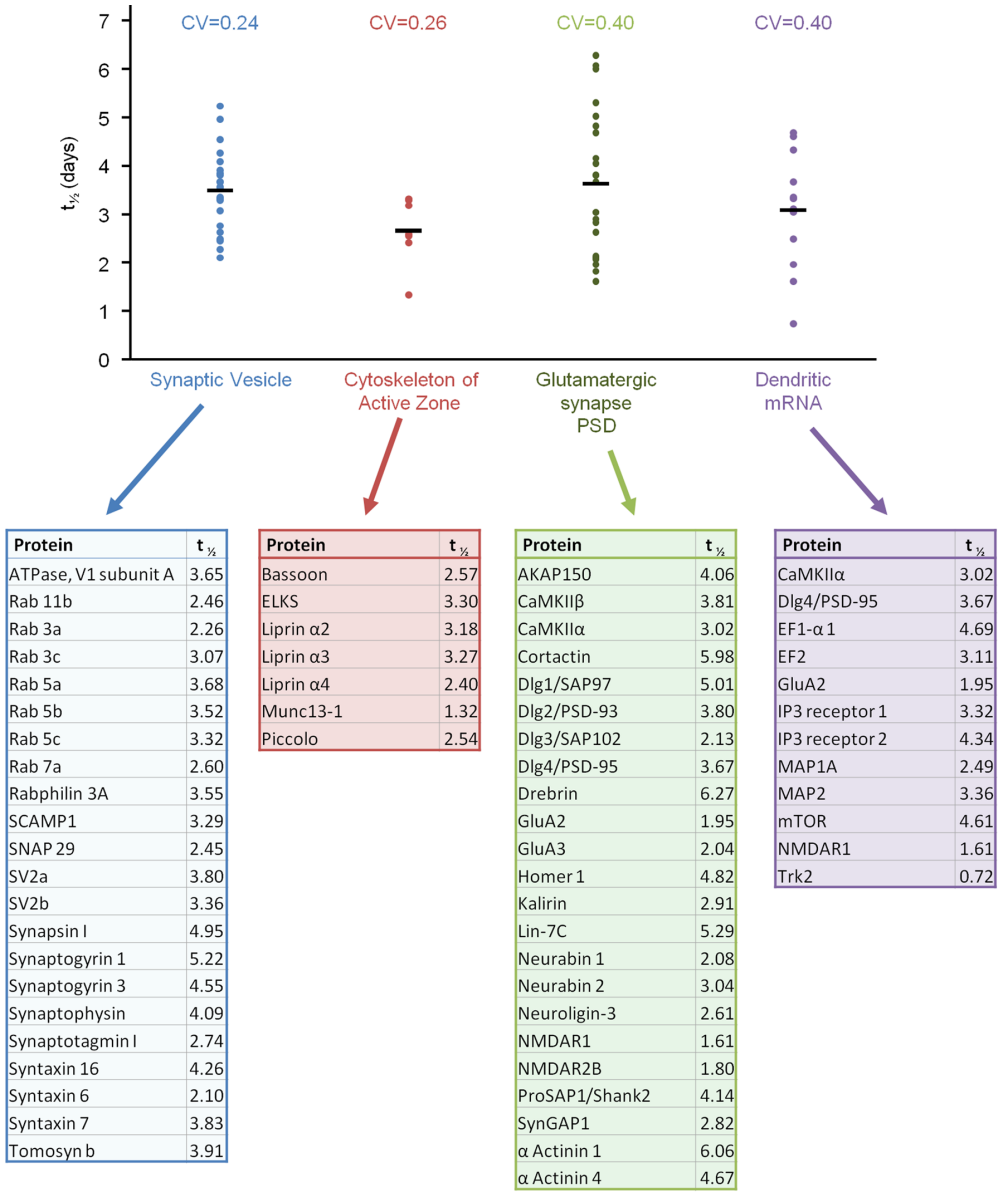
Comparisons of metabolic half-life estimates for proteins localized to particular synaptic compartments. Groups of well characterized proteins were curated manually and estimates of their metabolic half-lives were compared. Each dot represents the half-life value of one protein. Horizontal bars represent average values for each group. The coefficient of variation for each group is provided above each group. Proteins contained in each group along with estimates of their metabolic half-lives are listed below the graph. Except for the difference between the Synaptic Vesicle and Cytoskeleton of Active Zone groups (p = 0.01) all other differences between groups were not statistically significant (Kolmogorov-Smirnov test).

The unique architecture of neurons might be expected to impose constraints on protein turnover rates that differ from one neuronal compartment to another. For example, proteins of axonal presynaptic compartments, which are typically located at large distances from the biosynthetic machinery at the cell body, might be expected to undergo slower turnover than, for instance, postsynaptic proteins synthesized locally in dendrites. To examine this possibility we collated groups of well characterized proteins (Fig. 6): 1) presynaptic vesicle proteins [37]; 2) presynaptic active zone molecules [38], [39]; 3) postsynaptic density (PSD) proteins of glutamatergic synapses [40]; and 4) proteins for which dendritically located mRNAs are consistently found [41]–[51] (reviewed in [52]–[54]). We then determined whether metabolic turnover rates differed significantly among these groups. As shown in Fig. 6 the differences were surprisingly modest. Perhaps most unexpected was the finding that the half-lives of presynaptic active zone molecules (Piccolo, Bassoon, Munc13-1, ELKS, α-Liprins) and synaptic vesicle proteins (with and without transmembrane domains) were not significantly longer (if anything, they were shorter) than those of PSD proteins or proteins for which mRNAs are consistently found in dendrites. Interestingly, GO annotation analysis showed that the half-lives of proteins tagged as axonal were distributed in a manner similar to that of the entire population (Fig. S9C).

Comparisons of metabolic turnover rates for proteins that are structurally or functionally related seemed to indicate that in some cases, their metabolic turnover rates are also very similar. For example Piccolo and Bassoon, two huge presynaptic active zone proteins that share many properties, were found to have nearly identical metabolic turnover rates (half-lives of 2.54 and 2.56 days respectively; Fig. 3). Similarly, GluA2 and GluA3, the major subunits of AMPA-type glutamate receptors, exhibited very similar metabolic turnover rates (half-lives of 1.95 and 2.04 days respectively; Fig. 3). Such similarities are potentially interesting as they might indicate that the biogenesis and degradation of functionally related proteins may be coupled, perhaps at the level of functional complexes or subcellular organelles (fully assembled receptors, units of active zone material, synaptic vesicles, etc.). We therefore performed several in-depth analyses to examine this possibility in a systematic manner.

**Figure 7:**
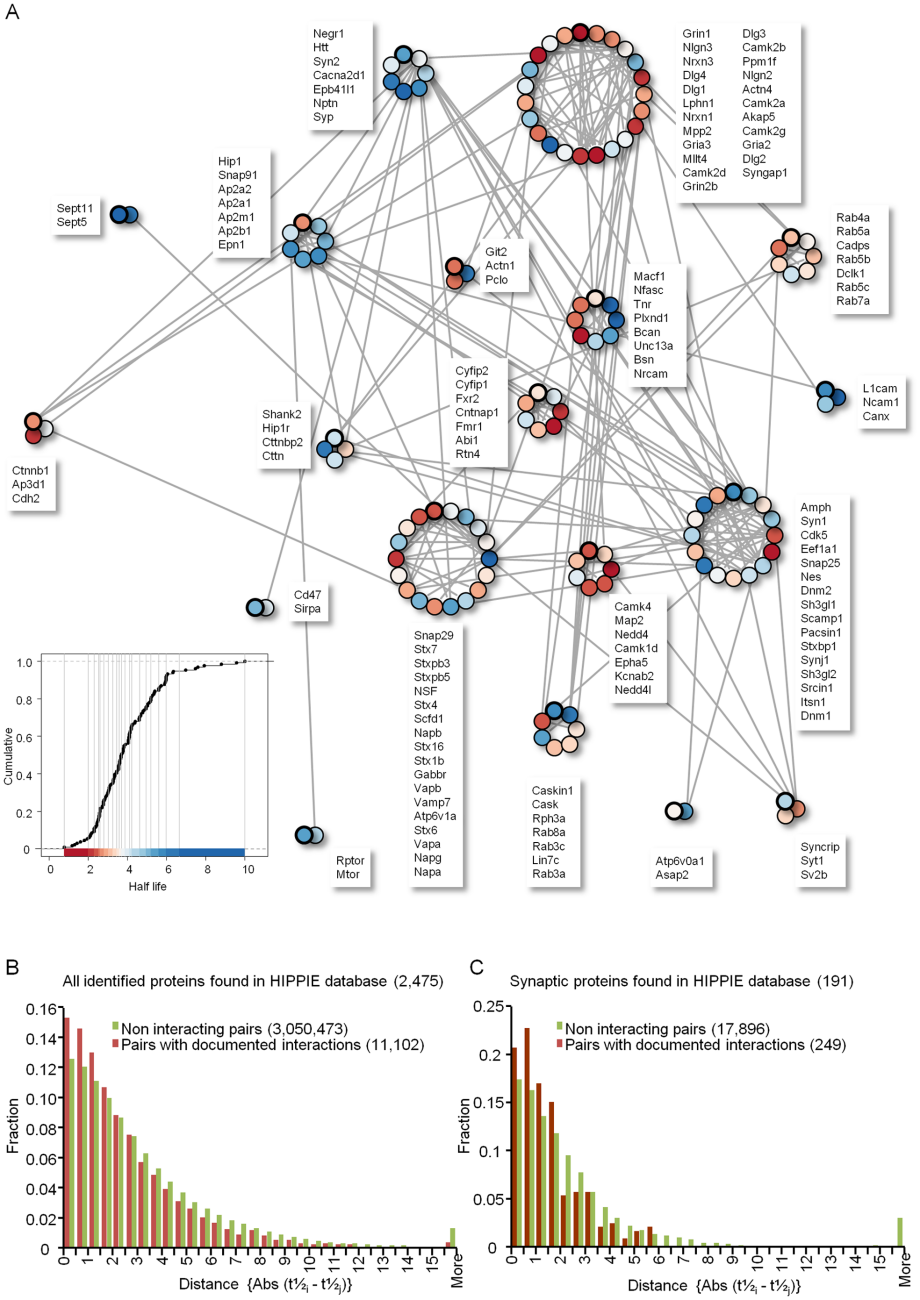
Relationships between half-life estimates and protein-protein interaction groups. **A)** A molecular interaction network of 191 synaptic related proteins (main text and Table 1) generated on the basis of a manually curated public domain protein-protein interaction database (Human Integrated Protein-Protein Interaction Reference, or HIPPIE; [55]; see Materials and Methods for further details). Each circle represents one protein, with the estimated metabolic half-life for that protein color coded according to the legend at the bottom left corner. Proteins in each cluster are listed in clockwise fashion, with the top protein in each list referring to the circle in each cluster encompassed with a thick line. **B,C)** Differences between metabolic turnover rates are smaller on average for pairs of interacting proteins as compared to pairs of non-interacting proteins. Absolute differences between metabolic half-life estimates for all pairs for which interactions are known to exist were compared to all pairs for which interactions are not known to occur (see main text for details), and the distributions of such differences were plotted for both groups. **B)** All identified proteins, and **C)** For the list of synaptic and synaptically related proteins. In both cases, differences between groups were highly significant (p ≪10^−10^, Kolmogorov-Smirnov test).

First we used protein-protein interaction databases to examine the hypothesis that proteins belonging to the same multimolecular complex will exhibit similar turnover rates. To that end, we generated a network from the 191 synaptic and synaptically related proteins mentioned above, based on a manually curated public domain protein-protein interaction database (Human Integrated Protein-Protein Interaction Reference or HIPPIE; [55]; see Materials and Methods for further details). The network was then “pruned” to include only proteins for which metabolic turnover rates were determined here and clustered (Fig. 7A; see Materials and Methods for details). ANOVA of the resulting clusters strongly indicated that the distributions of protein half-lives were not identical for all clusters (p<0.002; see Materials and Methods) indicating that relationships existed between membership in a cluster and protein half-life. To further explore such relationships, we compared the degree to which turnover rates within clusters were more similar to each other as compared to what might be expected by chance. Given the relatively small number of proteins in our list of synaptic and synaptically related proteins, this was performed on half-lives measured here for proteins listed in two published databases of synaptic proteins [56], [57] (see Materials and Methods for further details). This analysis revealed that for small clusters (containing 3 or 4 proteins), half-life estimates were often more similar than would be expected by chance (p<0.05; data not shown). Here too, ANOVA of all clusters indicated that distributions of protein half-lives were not identical for all clusters (p<0.05).

Using the same database (HIPPIE), we also performed a pairwise analysis, in which we compared the metabolic turnover rates of all protein pairs for which verified protein-protein interactions were listed, to all other pairs for which the same database did not contain strong evidence for such interactions. The comparison was based on absolute differences between the half-lives of two proteins *i* and *j* such that
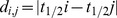



As shown in Fig. 7B, a comparison of half-life differences from 11,102 pairs with known interactions to 3,050,473 non-interacting pairs revealed that differences between metabolic turnover rates for protein pairs with known interactions were smaller (interacting: mean±SEM 2.58±0.025, median = 1.86; non-interacting: mean±SEM 3.26±0.002, median = 2.26; 2,475 proteins; p≪10^−10^, Kolmogorov-Smirnov test). An even greater difference emerged when this analysis was limited to the 191 proteins belonging to our synaptic protein list (Fig. 7C; interacting: mean±SEM 1.56±0.083, median = 1.21; non-interacting: mean±SEM 2.62±0.030, median = 1.63; 191 proteins; 249 interaction pairs, 17,896 non interaction pairs; p≪10^−10^, Kolmogorov-Smirnov test). Taken together, these analyses indicate that metabolic turnover rates of interacting proteins, and small protein groups belonging to particular multimolecular complexes, are, in some cases, more similar than would be expected by chance.

As mentioned above, we noted some conspicuous similarities in metabolic turnover rates for functionally related groups of synaptic proteins. The bioinformatic analyses described above might be taken to indicate that such similarities are not accidental. It is thus interesting to point out some further similarities. Beyond those mentioned already for the active zone molecules Piccolo and Bassoon, and for the AMPA receptor subunits GluA2 and GluA3 (which were also very similar to those of NMDA receptor subunits NR1 and NR2B), similar turnover rates were observed for 1) Neuroligin-2, Neuroligin-3, Neurexin-1 and Neurexin-3, proteins involved in important transynaptic interactions [58] (half-lives of 2.56, 2.60, 2.89, 2.61, days respectively); 2) Cortactin, α-Actinin and Drebrin – actin-binding proteins linked to dendritic spine regulation [59] (half-lives of 5.98, 6.06, and 6.27 days, respectively) and 3) PSD-95 and PSD-93 (half-lives of 3.67 and 3.80 days, respectively). It is also worth pointing out that half-life coefficients of variation for proteins tightly associated with synaptic vesicles and for active zone proteins were smaller than those of the other groups (Fig. 6), implying tighter distributions of metabolic turnover rates within these groups (see also Fig. 3E). This would be consistent with the possibility that the biogenesis/degradation of (some of) these components might be coupled via common trafficking intermediates [60].

### Protein Synthesis Load Imposed by the Need to Maintain Synapses

Recent studies have provided quantitative information on typical copy numbers of many synaptic proteins at individual synapses (reviewed in [40]) and synaptic vesicles [37], [61]. Combining this data with measurements of metabolic turnover rates allows for some estimation of the protein synthesis load that synapses impose on neurons and their biosynthetic machinery.

**Figure 8:**
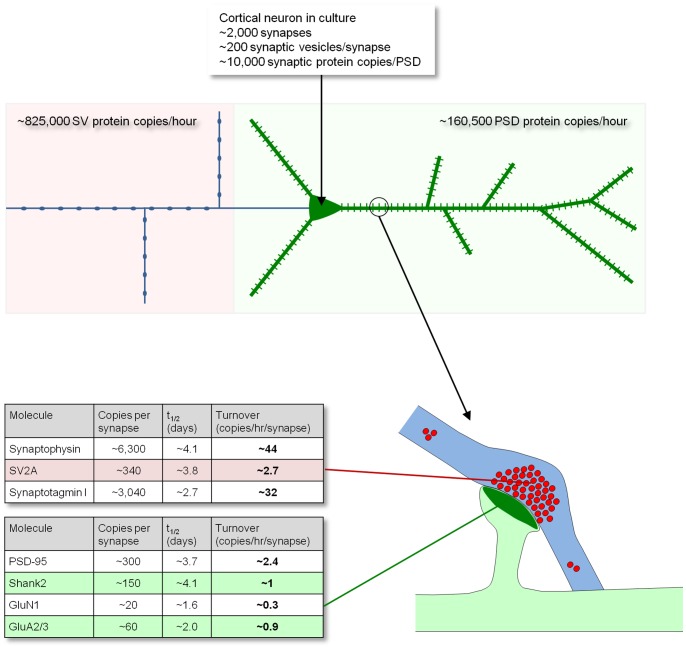
Metabolic load of synaptic protein synthesis. Schematic illustration of a neuron (top) and a synapse (bottom) with some estimates of protein synthesis rates required to maintain the synapse population of a prototypical cortical neuron in primary culture (top) or the synaptic contents of some specific molecules (bottom).

Reconstruction of neurons expressing a GFP-tagged variant of PSD-95 [33] (Fig. S11) allowed us to estimate the number of glutamatergic synapses made on the dendritic tree of individual cortical neurons in culture to be on the order of 2,000. Using this data, the aforementioned literature on synaptic protein copy number, the assumption that, on average, presynaptic boutons contain ∼200 synaptic vesicles [62] and the assumption that the number of presynaptic boutons an individual neuron has is, on average, identical to the number of synapses it receives, we calculated the daily synthesis rates of some key synaptic proteins in cultured cortical neurons (Fig. 8). For the most abundant synaptic proteins, these rates are very substantial. Thus for example, for the postsynaptic density protein PSD-95 (/SAP90/Dlg4; copies/synapse ≈300) synthesis rates are estimated to be ∼113,450 copies per day or ∼79 copies per min. For postsynaptic CaMKII (for which a figure of 5,600 copies/synapse has been reported [40]) synthesis rates are estimated to be ∼2,272,200 copies per day or ∼1,580 copies per min. Similarly, for the synaptic vesicle proteins Synaptophysin and Synaptotagmin 1 (copies/vesicle ≈31 and 15, respectively) synthesis rates are estimated to be ∼2,136,300 and 1,538,400 copies per day or ∼1,484 and 1,068 copies/min respectively.

If an average glutamatergic PSD contains ∼10,000 protein copies [40] and the average half-life of PSD proteins is ∼3.6 days (Fig. 6), then for a neuron with 2,000 excitatory synapses, the biosynthesis rate needed to maintain its postsynaptic densities is approximately 3,850,600 protein copies per day, or about 2,670 copies per minute – roughly equivalent to synthesizing the protein content of one PSD every four minutes. For maintaining the pool of synaptic vesicles, assuming that individual synaptic vesicles contain ∼250 protein copies [37] and that the average half-life of synaptic vesicle proteins is ∼3.5 days (Fig. 6), required biosynthesis rates are about 19,802,900 copies per day, or 13,800 copies per minute – equivalent to synthesizing the protein content of ∼55 synaptic vesicles every minute or, put differently, the synaptic vesicle content of one synapse roughly every four minutes. Assuming that all biosynthesis of presynaptic vesicle proteins occurs in the soma, the latter figure predicts that the protein equivalent of about 55 synaptic vesicles is trafficked each minute through the axon initial segment. Given a mean half-life for synaptic proteins of 4.14 days (Fig. 2B), a reasonable approximation would be to summarize that about 0.7% of the synaptic protein content of these neurons is turned over every hour. This estimate is in good agreement with the loss of <10% of synaptic immunofluorescence we observed following exposure to anisomycin for 10 hours (Fig. 5J).

This analysis indicates that maintaining the protein content of a neuron's synapse population places significant demands on its protein synthesis machinery. Given that this analysis was limited to a subset of synaptic proteins (ignoring presynaptic membrane and active zone proteins, calcium channels, and a multitude of non-exclusive synaptic proteins) the protein synthesis load imposed by synapses (in terms of protein copy number) is almost certainly much higher than our estimates suggest.

## Discussion

To gain a better understanding of relationships between protein turnover, synaptic maintenance and remodeling, we used dynamic SILAC, MS, FUNCAT, quantitative immunohistochemistry and bioinformatics to systematically measure the metabolic half-lives of synaptic proteins, to examine how these depend on their cellular localization and on association with particular molecular complexes, and to assess the metabolic load synaptic proteostasis places on individual neurons. In contrast with several recent studies in which half-lives of several hours were reported for a number of major synaptic proteins, we found that nearly all synaptic proteins identified here (191) exhibited half-lifetimes in the range of 2–5 days. Unexpectedly, metabolic turnover rates were not significantly different for presynaptic and postsynaptic proteins, or for proteins for which mRNAs are consistently found in dendrites. Some functionally or structurally related proteins exhibited very similar turnover rates, indicating that their biogenesis and degradation might be coupled, a possibility further supported by bioinformatics-based analyses. Our findings suggest that ∼0.7% of the synaptic protein content of the neurons studied here is turned over every hour, and that this turnover places a substantial load on the neuronal biosynthetic and transport mechanisms. The implications of these findings are discussed below.

### Methodological Considerations

Our estimates of protein half-lives were based on several assumptions, some of which warrant discussion.

First, we assumed that the total amount (H+L) of each protein species remained constant and thus, incorporation rates represented the complement of degradation rates. As we and others have noted, however, synapse numbers tend to increase at moderate rates during the one week period used here. Indeed, we measured a 27% increase in synaptic numbers over this period (Fig. S5A,B). Consequently this would lead to a small overestimation of actual turnover rates, as illustrated in Fig. S5C,D. On the other hand, the fraction of synaptic proteins in neuronal extracts barely increased during this period (Fig. S6) and no significant differences were observed when turnover rates during the third week and fourth weeks *in vitro* were compared (Fig. S7). We therefore surmise that the modest growth in synaptic numbers did not severely affect our half-life estimates and in any case would not result in underestimates of turnover rates.

Second, we assumed that heavy AA incorporation would follow single exponential kinetics. While Figs. 1C, 3A–D and S3 generally support this assumption, in a minority of cases fits to single exponential functions were less than perfect. This might be due to several reasons: 1) H/L ratio inaccuracies (due to peptide identification or quantification errors); 2) Multiple pools of protein species that differ greatly in their turnover rates; 3) Changes in turnover rates over the seven-day experiment period. Proteins for which fits were very poor were excluded (2%), but for some of the remaining identified proteins (∼6%; Fig. S3) fits were imperfect and thus estimates for these proteins might be less accurate than desirable.

Our third assumption was that the majority of synaptic proteins subjected to MS analysis originated from synaptically localized pools. Immunolabeling here (Fig. 5, Fig. S5A) and elsewhere indicates that in these preparations, the majority of PSD, CAZ and synaptic vesicle proteins are localized to synaptic junctions. As mentioned in the introduction, however, imaging studies indicate that synaptic proteins continuously move between synaptic and extrasynaptic pools [18]–[24] at rates that greatly exceed their metabolic turnover rates (see below). These dynamics imply that the distinction between synaptic and extrasynaptic pools becomes blurred over the time scales of these experiments (days) and thus metabolic turnover rates measured here probably represent some combination of turnover rates for synaptic and extrasynaptic proteins.

One last point that deserves explicit mention is that the cell culture preparations used here contained glial cells. Thus, for proteins that are not specific to neurons, reported rates reflect a combination of turnover rates in neurons and glia.

### Implications for Synapse Biology

To obtain a realistic understanding of synaptic maintenance and to constrain hypotheses regarding relationships between synaptic plasticity and protein synthesis/degradation, reliable estimates of synaptic protein metabolic turnover rates are essential. To date, few attempts have been made to systematically measure such rates. One notable study is that of Ehlers (2003) [3] in which ^35^S pulse-chase labeling was used to measure metabolic turnover rates in cultured rat cortical neurons. The study reported an average t_½_ for total PSD proteins of 3.25 hours (τ = 4.7 hours) and an average t_½_ for 10 specific PSD proteins of <10 hours (τ ≈13.5 hours). The half-lives reported for these proteins were much shorter than those reported here {for example NR1∶13 h vs. 38 h; Dlg3/SAP102∶7 h vs. 51 h; Dlg4/PSD-95∶8 h vs. 88 h; CaMKIIβ-2∶14 h vs. 91 h; Ehlers (2003) [3] vs. current data, respectively}. It is important to note, however, that the relatively short (12 h) “pulse” period used in the aforementioned study would strongly bias estimates towards pools with fast turnover rates, because proteins and protein pools with slow turnover rates would barely become labeled and thus, would be underrepresented in subsequent chase periods. Indeed, our FUNCAT experiments, in which the pulse duration was 24 hours, resulted in somewhat shorter estimates of global protein turnover rates (Fig. 4) as compared to those obtained in our SILAC experiments in which the “pulse” period was much longer (7 days). As a result, our SILAC experiments were probably less biased toward protein pools with rapid metabolic turnover rates.

In a more recent study [63] organism-wide isotopic labeling and MS were used to compare protein turnover rates in mouse brain, liver and blood tissues. Labeling was achieved by providing adult mice with a diet supplemented with ^15^N-enriched algae. Unlike dynamic SILAC, where labeled AA introduction is abrupt and temporally well defined, labeling in that approach is protracted and much less controlled. Consequently, turnover estimations require corrections for AA ingestion, excretion, internal metabolism and catabolism kinetics. Nevertheless, it is interesting to compare estimates of turnover rates for synaptic proteins identified in that study to those obtained here. As a rule, these estimates were much slower (e.g. Synaptophysin: 502 h vs. 98 h; Bassoon: 240 h vs. 62 h; GluA2∶173 h vs. 47 h; Dlg4/PSD-95∶367 h vs. 88 h; CaMKIIβ-2∶157 h vs. 91 h; [63] vs. current data, respectively). Perhaps here too, the longer “pulse” period (32 days) exposed additional pools with even slower turnover rates (see [64]). Assuming these differences are not due to methodological issues, this study might indicate that turnover rates of synaptic proteins in adult mice are even slower than those reported here (compare also [65] and [66], to [67] for AMPA receptor subunits). Interestingly, a comparison with protein turnover in HeLa cells [32] shows that protein turnover in primary cultures of cortical neurons is generally slower than that observed in this immortalized cell line.

It is generally thought that the bulk of synaptic proteins is synthesized in the cell body and thereafter transported to synaptic sites. The transport of many synaptic proteins can be rather slow (millimeters/day; [5]–[8]). The relatively slow turnover rates reported here are quite compatible with such transport rates. On the other hand, if synaptic protein turnover is as rapid as previously suggested for PSD proteins (such as PSD-95; t_½_≈8 h [3]) or for the presynaptic active zone protein Rim1 (t_½_≈0.7 h; [4]), some way of reconciling rapid turnover with slow transport is called for [16]. It is worth noting that in our hands, synaptic Rim and PSD-95 levels were reduced by only ∼8% and 10% following 10 h exposures to anisomycin (Fig. 5G,I), in better agreement with an estimate of El-Husseini and coworkers [68] for PSD-95 (t_½_≈36 h).

Discrepancies between turnover and trafficking rates might possibly be resolved by reconsidering the roles of local protein synthesis in dendrites [9]–[12] and axons [13], [14], [16]. Although these are usually discussed in the context of synaptic plasticity, perhaps their primary role is to maintain the synaptic contents of (remote) synapses [16]. In this respect it is interesting to note that no major differences were observed in the average turnover rates of presynaptic (axonal) or postsynaptic (dendritic) proteins, in spite of their very different distances from the cells' major biosynthetic center, i.e. the soma, nor were these different for proteins for which synthesis is assumed to occur in dendrites (Fig. 6). Given the relatively slow turnover rates of synaptic proteins reported here, local protein synthesis rates need not be very high. At the extreme, if polyribosomes located near spines (e.g. [69], [70]) are to synthesize the entire protein contents of 2–8 PSDs (∼20,000 to 80,000 molecules [40]), and if the average half-life of postsynaptic proteins is ∼3.5 d, synthesis rates would need to be ∼240 to ∼960 copies/hour or ∼4 to ∼16 copies/min. More realistically, however, local synthesis might mainly be important for supplying proteins with relatively high turnover rates, such as glutamate receptor subunits (Fig. 3; see [48]).

As mentioned above, live imaging studies based on fluorescence recovery after photobleaching (FRAP), photoactivation and single particle tracking consistently suggest that synaptic molecules continuously move into, out of and between synapses at fairly rapid rates (reviewed in [18]–[24]). In comparison to metabolic half-lives, residency half-lives are orders of magnitude shorter (Synapsin-1: ∼1 h vs. ∼4.9 days; Bassoon: ∼3 h vs. ∼2.6d, Munc-13–1: ∼1 h vs. ∼1.3d; PSD-95: ∼3.3 h vs. ∼3.7d; residency vs. metabolic turnover half-lives, respectively [7], [71], [72], [73]); these differences become even more apparent when considering the short residency times of integral membrane proteins (e.g. [22], [24], [74]). The predominance of exchange rates over metabolic turnover rates [7] would seem to have two fundamental implications: First, it would seem to suggest that the availability of many, if not most, synaptic proteins is not a limiting factor when rapid changes in synapse composition and size are required, simply because synaptic components can be recruited from nearby synapses, in a manner similar to that observed during synaptogenesis (e.g. [75]–[77]). Second, it would seem to question the ability of local synthesis and degradation processes to regulate the composition of individual synapses in isolation from neighboring synapses, because proteins added to one synapse might migrate to neighboring ones. It should be noted, however, that the generality of protein exchange predominance over metabolic turnover is yet to be determined, given that some dendritic proteins exhibit higher metabolic turnover rates (e.g. Fig. 4, [35]). It is possible that such proteins escaped detection here due to, for example, relatively low abundance [78]. Perhaps scarce proteins, synthesized locally, play crucial roles in determining synaptic properties on time scales that are faster than protein exchange rates [79], [80]. We expect that future studies, using new techniques to identify and localize newly synthesized proteins [81], will gradually clarify the physiological relationships between protein exchange, protein turnover, and synaptic plasticity.

## Materials and Methods

### Ethics

All experiments were performed in primary cultures of rat neurons prepared according to a protocol approved by the “Technion, Israel Institute of Technology Committee for the Supervision of Animal Experiments” (ethics approval number IL-099-08-10).

### Cell Culture

Primary cultures of rat cortical neurons used for SILAC experiments were prepared as described previously [33]. Briefly, cortices of 1–2 days-old Wistar rats of either sex were dissected, dissociated by trypsin treatment followed by trituration using a siliconized Pasteur pipette. 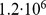
cells were then plated in 12-well plates whose surface had been pretreated with polyethylenimine (Sigma) to facilitate cell adherence. Cells were initially grown in medium containing minimal essential medium (MEM; Sigma), 25 mg/l insulin (Sigma), 20 mM glucose (Sigma), 2 mM L-glutamine (Sigma), 11.16 mg/l gentamycin sulfate (Sigma), 10% NuSerum (Becton Dickinson Labware), and 0.5% fetal bovine serum (HyClone). The preparation was then transferred to a humidified tissue culture incubator and maintained at 37°C in a 95% air and 5% CO_2_ mixture. Half the volume of the culture medium was replaced three times a week with feeding medium similar to the medium described above but devoid of NuSerum, containing a lower concentration of L-glutamine (Sigma, 0.5 mM), and 2% B-27 supplement (Gibco).

Primary cultures of rat hippocampal neurons for protein synthesis inhibition experiments were prepared as described previously [7]. In brief, hippocampal CA1–CA3 regions of 1–2 days-old Wistar rats of either sex were dissected and dissociated as described above and plated onto 22×22 mm coverslips coated with poly-D-lysine (Sigma) inside 8-mm-diameter glass cylinder (Bellco Glass) microwells. Culture medium consisted of minimal essential medium (MEM; Gibco), 20 mM glucose, 0.1 g/l bovine transferrin (Calbiochem), 25 mg/l insulin (Sigma), 2 mM L-glutamine (Sigma), 10% NuSerum (Becton Dickinson Labware), 0.5% fetal bovine serum (HyClone), 2% B-27 supplement (Gibco), and 8 µM cytosine β-D-arabinofuranoside (Sigma) which was added to the culture medium after 3 d. Cultures were maintained at 37°C in a 95% air and 5% CO_2_ humidified incubator. Culture medium was replaced every 7 d.

Primary cultures of rat hippocampal neurons for FUNCAT (FlUorescent Non–Canonical Amino acid Tagging) experiments were essentially prepared as described previously [82]. Cells were prepared from E18 brains of Wistar rats (either sex), raised in the animal facility of the Leibniz Institute for Neurobiology, dissociated in Ca2+ and Mg2+ free Hank’s balanced salt solution with 0.25% Trypsin and plated on Poly-D-lysine coated glass coverslips at desired densities in DMEM including 10% fetal calf serum, 2 mM glutamine, and antibiotics (100 U/ml penicillin, 100 µg/ml streptomycin, 250 ng/ml Fungizone®) (all Invitrogen) and kept in humidified atmosphere at 37°C and 5% CO_2_. The medium was replaced 24 h later with Neurobasal medium supplemented with B27, 0.8 mM glutamine and antibiotics.

### SILAC

After cortical cells were grown for 14 days, media containing heavy isotope-labeled variants of lysine {Lys8, (^13^C_6_, ^15^N_2_)} and arginine {Arg10, (^13^C_6_, ^15^N_4_)} (Cambridge Isotope Laboratories) was added to the culture dishes, resulting in an excess of heavy lysine and arginine (5∶1) as compared with the non-labeled variants of these amino acids. Neurons were harvested immediately (time 0) and after 1, 3, and 7 days. For preparations maintained for 3 and 7 days before harvesting, the feeding media used contained heavy lysine and arginine at concentrations that preserved the 5∶1 heavy to light (H/L) ratios. Harvesting was done by gently washing the cells 3 times in a physiological solution (“Tyrode’s”, 119 mM NaCl, 2.5 mM KCl, 2 mM CaCl_2_, 2 mM MgCl_2_, 25 mM HEPES, 30 mM glucose, buffered to pH 7.4), aspirating all the solution, and immediately adding 100 µl of lysis buffer composed of 10% sodium dodecyl sulfate (SDS; Sigma), 30 mM TRIS HCl (Sigma), 3.4% glycerol, 25 mM DTT (Sigma), and 0.5% v/v protease inhibitor (Calbiochem). The cells were then scraped in the lysis buffer using a disposable cell scraper. The lysate was then collected, pipetted vigorously on ice, boiled for 5 min, and frozen at −80°C until used.

For “conventional” SILAC experiments, the cells were grown in lysine and arginine-free MEM (Biological Industries) to which Lys8 and Arg10 were added (0.4 mM and 0.6 mM respectively). After two weeks, cells were gently washed by partial replacement of media with lysine and arginine-free media to which lysine and arginine (Sigma) were added at identical concentrations. Following 3 washes, conditioned media from “sister” preparations (grown in media containing light AAs which were never exposed to heavy AA) was added. Cells were harvested and processed at days 0, 1, 3, and 7 as described above.

### Labeling Newly Synthesized Proteins, FUNCAT and Immunocytochemistry

The medium of primary cultures hippocampal neurons prepared as described above (21 days in culture) was exchanged to methionine-free Hibernate-medium [83] supplemented with B27, 0.8 mM glutamine, antibiotics (100 U/ml penicillin, 100 µg/ml streptomycin, 250 ng/ml Fungizone®) and 4 mM AHA (prepared according to [84]). Cells were either washed with PBS-MC (1 mM MgCl_2_, 0.1 mM CaCl_2_) at pH 7.4 and fixed with 4% paraformaldehyde in PBS for 5 min at room temperature or cultivated in methionine-free Hibernate-medium supplemented with B27, 0.8 mM glutamine, antibiotics and 4 mM methionine and fixed after 24 h or 48 h. The FUNCAT procedure was carried out as described previously [35] with minor changes. Fixed cells were incubated in B-block solution (10% horse serum, 5% sucrose, 2% bovine serum albumin, 0.1% Triton X-100) for 1.5 h and washed three times for 10 min with PBS pH 7.8. Coverslips were incubated in a “click-reaction”-mixture composed of 200 µM TBTA, 500 µM TCEP and 200 nM Tetramethylrhodamine (TAMRA)-alkyne-tag (Invitrogen) upside-down protected from light at room temperature overnight. Coverslips were washed as described [35] and incubated with primary antibodies diluted in B-block solution for 2 h at room temperature, washed three times with PBS pH 7.4 and incubated subsequently with Alexa-488 or Cy5-conjugated secondary antibodies in B-block solution for 1 h at room temperature. Mounting was done using Mowiol-solution (Calbiochem). The following antibodies were used for immunocytochemistry staining in concentrations as indicated: MAP2 (1∶1000, Sigma-Aldrich), Synaptophysin (1∶2000, SYnaptic SYstems). Fluorescently labeled secondary antibodies (Jackson Laboratories) were used as follows: Alexa 488 conjugated anti-rabbit (IgG, 1∶1000), Cy-5 conjugated anti-guinea pig (IgG, 1∶1000).

### Immunolabeling Against Synaptic Proteins Following Protein Synthesis Inhibition

At 14 days in culture, primary hippocampal cultures prepared as described above were treated with 25 µM anisomycin (Sigma) or vehicle. The cells were fixed after 10 h, and stained using antibodies for synaptic proteins. As a positive control we examined the effect of anisomycin application on c-fos expression. To that end, cells were treated with 25 µM of anisomycin or vehicle only for 2 h and then either exposed to 10 µM glutamate or not (as these neuronal networks are spontaneously active) for 2 h. The cells were then fixed and stained against c-fos. The fraction of cells in which bright nuclear c-fos immunolabeling was visible was then quantified.

Immunolabeling was performed by washing the cells in Tyrode's solution followed by fixation with 4% paraformaldehyde (PFA) and 120 mM sucrose in PBS (fixative solution) for 20 min, or cold (−18°C ) methanol for 5 min. PFA-fixed cells were permeabilized for 10 min in fixative solution to which 0.25% Triton X-100 (Sigma) was added. The cells were washed three times in PBS, incubated in 10% bovine serum albumin (BSA, Sigma) for 1 h at 37°C, and incubated overnight at 4°C with primary antibodies in PBS and 1% BSA. The cells were then washed three times for 5 min with PBS and incubated for 1 h at room temperature with secondary antibodies in PBS and 1% BSA. The cells were washed again with PBS three times, and imaged immediately.

Primary antibodies included: mouse anti-synapsin I 1∶400 (TransLabs); mouse anti-PSD-95, clone 108E5 1:150 (Synaptic Systems); mouse anti-PSD-95, 1∶500 (Upstate); rabbit anti-SV2A 1∶500 (Synaptic Systems); monoclonal anti-bassoon 1∶400 (a generous gift of Craig Garner, Stanford University); guinea-pig anti-ProSAP2 1:800; rabbit anti-ProSAP1 1:2000 (both generous gifts of Tobias M. Boeckers, Ulm University, Germany); rabbit anti-piccolo 1∶200 (a generous gift of Dr. Wilko Altrock, Leibniz Institute for Neurobiology, Magdeburg, Germany); mouse anti-RIM 1∶200 (BD Transduction Laboratories); rabbit anti-VGAT (Synaptic Systems) 1∶200; rabbit anti-c-fos antibody 1∶400 (sc-52, Santa Cruz). Secondary antibodies included: AlexaFluor 633 nm goat anti-mouse IgG2a (Invitrogen), Cy5 donkey anti-mouse (Jackson ImmunoResearch Laboratories), Cy5 donkey anti-guinea pig (Jackson ImmunoResearch Laboratories), Cy5 donkey anti-rabbit (Jackson ImmunoResearch Laboratories). All secondary antibodies were used at a dilution of 1∶200.

### In Gel Proteolysis and Mass Spectrometry Analysis

48 µg of protein from each time point were separated on 7% SDS-PAGE (Polyacrylamide Gel Electrophoresis; two lanes for each time point) and sliced into 9 sections, including the stacking gel as shown in Fig. 1A. The proteins in each gel slice were reduced with 2.8 mM DTT (60°C for 30 min), modified with 8.8 mM iodoacetamide in 100 mM ammonium bicarbonate (in the dark, room temperature for 30 min) and digested in 10% acetonitrile and 10 mM ammonium bicarbonate with modified trypsin (Promega) overnight at 37°C.

The resulting tryptic peptides were resolved by reverse-phase chromatography on 0.075 X 200-mm fused silica capillaries (J&W) packed with Reprosil reversed phase material (Dr Maisch GmbH, Germany). The peptides were eluted with linear 95 minute gradients of 7 to 40% and 8 minutes at 95% acetonitrile with 0.1% formic acid in water at flow rates of 0.25 µl/min. Mass spectrometry was performed by an ion-trap mass spectrometer (Orbitrap, Thermo) in a positive mode using repetitively full MS scan followed by collision induced dissociation of the 7 most dominant ions selected from the first MS scan.

The mass spectrometry data were analyzed using MaxQuant 1.2.2.5 (Max-Planck Institute for Biochemistry, Martinsried, Germany [29]) searching against the Rattus section of the NCBI-NR database with a false discovery rate of 1%. H/L ratios for all peptides belonging to a particular protein species were pooled, providing an average H/L ratio for each protein.

### Data Analysis

Data from two full four time point experiments and two single time point repeats (t = 3d) were pooled, with consolidation based on GI entries. For each protein at each time point, a weighted average of H/L ratios was calculated, with weights based on the number of peptides identified in each repeat. H/L ratios (*H_t/_L_t_*) for all time points (*t*) were converted into fractional incorporation ratios (*Ft*) and corrected to the maximal expected ratio *F_max_* ≈ 5/(5+1) according to
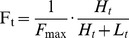



The data were then fit to exponential curves such that
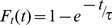
with τ representing the time constant of metabolic protein turnover. τ values were converted to half-life (*t_½_*) as follows *t_½_ = ln(2)· τ*. Proteins for which at least 1 peptide was identified from each of the four time points were included in the analysis. Goodness of fit to exponential curves was judged by the sum of square errors (SSE) values (Fig. S3). Proteins for which SSE >0.1 were excluded from further analysis (∼2%). For the set of synaptic proteins, the exclusion threshold was set to SSE>0.08. Analysis was done using Matlab (Mathworks) and Microsoft Excel. For Gene ID conversion, BioDBnet (http://biodbnet.abcc.ncifcrf.gov/ [85]) was used.

### Microscopy and Image Analysis

Imaging of immunolabeled neurons in protein synthesis inhibition experiments was performed using a custom designed confocal laser scanning microscope [7] using a 40X, 1.3 NA Fluar objective. Excitation was performed at 633 nm (Helium Neon Laser). Fluorescence emissions were read using a 650 nm long-pass filter (Semrock). Images were collected by averaging six frames at two to four focal planes spaced 0.8 µm apart. All data were collected at a resolution of 640×480 pixels, at 12 bits per pixel. Image analysis was performed using custom written software (OpenView) written by N.E.Z. Analysis was performed on maximal intensity projections of 2 sections, located 0.8 µm apart. Intensities of fluorescent puncta were measured by programatically centering 9×9 pixel regions of interest obtaining the average fluorescence intensity in each area as shown in Fig. 5F. Analysis of somatic immunofluorescence was performed using NIH ImageJ by manually placing regions of interest on somata, excluding the nuclei.

Images of immunolabeled neurons in FUNCAT experiments were acquired using a Zeiss Observer.Z1 microscope and the AxioVision 4.8 software. Image acquisition and image processing were performed with identical exposure times and settings for each treatment group within one experiment. Images were processed with NIH ImageJ. For quantification of synaptic fluorescence using OpenView, proximal dendritic segments were selected with a fixed length of 30 µm for all images analyzed.

### Recordings of Network Activity

Cortical neurons were plated on thin glass multielectrode array (MEA) dishes at densities identical to those used for SILAC experiments (see above). MEA dishes used here contained 59, 30 µm diameter, electrodes arranged in an 8×8 array, spaced 200 µm apart. The dishes were covered by a custom designed cap containing a submerged platinum wire loop serving as a ground electrode, heated to 37°C, and provided with a filtered stream of 5%CO_2_ and 95% air through an inlet in the cap. Network activity was recorded through a commercial 60-channel headstage/amplifier (Inverted MEA1060, MCS) with a gain of 1024× and frequency limits of 1–5000 Hz. The amplified signal was further amplified and filtered using a bank of programmable filter/amplifiers (Alpha-Omega, Nazareth, Israel), multiplexed into 16 channels, and then digitized by two A/D boards (Microstar Laboratories, WA, U.S.A.) at 24 KSamples/sec per channel. Data acquisition was performed using AlphaMap (Alpha-Omega). All data was stored as threshold crossing events with the threshold set to −20 µV. Electrophysiological data were imported to Matlab (MathWorks, MA, USA) and analyzed using custom written scripts.

### Bioinformatics

Protein interaction networks were generated using a public domain protein-protein interaction database (HIPPIE; [55]). The network was then “pruned” to include only proteins for which metabolic turnover rates were determined here and clustered using the edge betweenness scoring algorithm (R package Igraph; http://igraph.sourceforge.net/screenshots2.html). Single factor ANOVA of the resulting clusters (size 2 and up) was performed for both the original half-life estimates and for the logarithms of the values to correct for the skewed (non-normal) distribution of half-life estimates. To examine the degree to which turnover rates within clusters were more similar to each other as compared to what might be expected by chance, 1000 randomized networks of identical architecture but with shuffled turnover rates were analyzed in parallel to the original network. The number of clusters and their composition remains the same for all the networks while the distribution of the values for turnover rates varies. The width of the distribution for those values within a particular cluster was estimated as 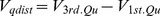, where 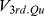 and 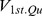
correspond to the values for turnover rates for the 3^rd^ and 1^st^ quartiles, respectively. A Kolmogorov-Smirnov test was performed to compare the obtained 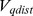 for clusters of equal size for the original network and for 1000 networks with shuffled values.

## Supporting Information

Figure S1 **Comparison of MS profiles of labeled and unlabeled samples.** Cortical neurons were either grown in the presence of 6× heavy AAs for 3 days (starting at day 14 *in vitro* as described in main text and in Materials and Methods) or maintained in standard growth media for the same period (control). Lysates of these preparations were then subjected to MS analysis as described in Fig. 1A. Data are from two duplicates, each subjected to separate MS analysis. **A)** Gel used to separate proteins according to molecular weight (stained with Coomassie Blue; gel image cropped to remove empty lanes). Two lanes were run for each sample to increase protein amounts. Gels were then sliced as in Fig. 1, proteins in each slice were digested, and resulting peptides from each slice and each duplicate were submitted separately to MS analysis. **B,C)** Number of peptides identified for each protein. Region in gray box is enlarged in bottom panel. Lines represent linear regressions. Data is the sum of peptide numbers identified for each protein in each duplicate. **D,E)** Total intensities of peptides identified for each protein. Region in gray box is enlarged in bottom panel. Lines represent linear regressions. Data is the average of intensities measured in duplicates. An excellent correlation was observed between MS profiles of heavy AA treated and control preparations. The deviations of the slopes from 1.0 are well within the range observed when comparing two samples. For example, slopes were 1.09 and 1.00 (peptides and intensities, respectively) for control-control duplicate comparisons and 1.15 and 1.34 for heavy AA- heavy AA duplicate comparisons. These differences probably reflect slight differences between the amounts of proteins loaded on the polyacrylamide gels.
(TIF)

Figure S2 **Effects of an excess of heavy AA on network activity.** Cortical neurons were grown on multielectrode (MEA) substrates for two weeks in an identical fashion to preparations used for SILAC experiments. The preparations were then mounted on an MEA amplifier as described previously [33]. Spontaneous activity was measured for 3 hours after which heavy AA (Heavy) or standard growth media (Control) were added to the MEA dish in a fashion identical to that performed in SILAC experiments. Recordings were then continued for another 12 hours. Action potentials (spikes) measured from all 60 electrodes were accumulated at 1 min intervals, the resulting spike rates were averaged over one hour time windows and normalized to mean spike rates during first 3 hours. A large variability was observed between networks, but the addition of heavy AA acids did not seem to have any particular effects.
(TIF)

Figure S3 **Distribution of sum of square errors (SSE) for fits to single exponentials.** SSE values for all identified proteins for which data was obtained for all four time points (2,859 proteins). The lower the SSE value, the better the fit. Note that the fit for the vast majority of proteins was excellent (SSE <0.02) and only a very small number of proteins (∼2%) exhibited unacceptable fits (SSE >0.1).
(TIF)

Figure S4 **Repeatability of half-life estimations. A)** Distributions of half-life estimates obtained separately in two experiments carried out two weeks apart. Only proteins for which data from all 4 time points was obtained in both experiments were included (1,622 proteins). **B)** Comparison of half-life estimates for individual proteins (1,608). Proteins with half-life estimates exceeding 20 days were excluded. **C)** Enlargement of region enclosed in gray box in B.
(TIF)

Figure S5 **Quantification of synaptic densities at the four time points of the SILAC experiments.** Cortical neurons were grown on glass bottom substrates for two weeks in an identical fashion to preparations used for SILAC experiments. The neurons were then fixed after an additional 0, 1, 3 or 7 days and stained against the PSD molecule PSD-95. Nine Z sections were then collected at 0.8 µm intervals at 14 to 17 fields of view in each dish. Synapses were counted programmatically as shown in Fig. 5 in sections #2 or #3 and #6 or #7 and counts were summed, and expressed as average count/field of view for all fields of view in two separate experiments. **A)** Two representative sections and the maximal intensity projection of all 9 sections. Images were taken at 17 days *in vitro* (i.e., two weeks +3 days in culture). Bar, 10 µm. **B)** Changes in synaptic density over the one week period. A mean growth of ∼4%/day in synaptic density was measured. **C)** Illustration of the effect that increases in total amounts of a given protein will have on half-life estimates. Note that the *relative* fraction of preexisting protein will seem to decrease (right panel) even though it does not change relative to the left panel. **D)** The anticipated effect of a 4% growth/day on the half-life estimate for a protein whose “real” half-life is 3.5 days. As shown here, this will lead to an apparent half-life of ∼3.0 days, that is, an overestimation of the turnover rates for this protein.
(TIF)

Figure S6 **Fractional intensity of synaptic proteins in SILAC experiments.** The intensity measured for all peptides (H+L) for all synaptic proteins, divided by the intensities measured for all identified proteins. As equal amounts of proteins were loaded on the separation gels, these data indicate that the fraction of synaptic proteins in the protein mixture barely changed over the 7-day experimental period, arguing against a large increase in synaptic numbers during this period.
(TIF)

Figure S7 **Comparison of fractional incorporation ratios for neurons maintained in culture for 2 weeks and 3 weeks.** Cortical neurons were maintained in culture for either 2 weeks or 3 weeks and then exposed to heavy AA for 3 days as described in main text and in Materials and Methods. Lysates of these preparations were then subjected to MS analysis as described in Fig. 1A. The fractional incorporation values were then compared for each protein identified in both data sets (2,460 proteins). Note the good correlation between the two data sets and the fact that the slope is ∼1.0.
(TIF)

Figure S8 **Distributions of metabolic half-life estimates obtained by “conventional” dynamic SILAC.** Neurons were grown for 2 weeks in lysine and arginine-free MEM to which Lys8 and Arg10 were added at nominal concentrations. After two weeks, cells were washed and placed in conditioned media from “sister” preparations (see Materials and Methods for details). 0, 1, 3, and 7 days later cells were lysed and processed as described above and half-life estimates were obtained from H/L ratios at 4 time points. **A)** Distribution of half-life estimates for all proteins for which H/L ratios were obtained at all 4 time points. Only proteins whose fits to single exponentials were satisfactory (SSE <0.1) were included. **B)** Comparison of half-life estimates for individual proteins for which data was obtained in both forms of dynamic SILAC experiments (Light→Heavy = data of Figs. 1–3; Heavy → Light = data of panel A).
(TIF)

Figure S9 **Distributions of metabolic half-life estimates of proteins selected according to particular GO annotations.** Subsets of proteins were selected according to specific GO annotations and distributions of their metabolic half-life estimates were compared to those of the entire population (2,804 proteins). **A)** Cell compartment: “Synapse” (105 proteins). **B)** Cell compartment: “Mitochondrial part” (240 proteins); **C)** Cell compartment: “Axon” (75 proteins).
(TIF)

Figure S10 **Enrichment analysis of proteins longest and shortest metabolic half-life estimates.** Lists of all neuronal proteins for which satisfactory half-life estimates were obtained were sorted according to their half-life estimates and subjected to GO based enrichment analysis (according to cellular component) using the public domain tool “GORILLA” (Gene Ontology enRIchment anaLysis and visuaLizAtion tool; [36]; http://cbl-gorilla.cs.technion.ac.il/). Note that enrichment analysis performed by this tool is based only on rank order, not absolute values. **A)** Enrichment analysis of proteins with longest metabolic half-life estimates. **B)** Enrichment analysis of proteins with shortest metabolic half-life estimates. The statistical significance of enrichment scores is color coded according to the index on the right hand side.
(TIF)

Figure S11 **Synaptic counts of individual cortical neurons in primary culture.** Cortical neurons expressing GFP-tagged PSD-95 grown on MEA dishes were used to quantify the number of excitatory synapses (bright dots) formed on cultured cortical neurons plated at the same densities as the preparations used for SILAC experiments. The neurons were imaged at days 19–20 in culture. Top image is from [33]. Bars, 50 µm.
(TIF)

Table S1 **List of 2,802 proteins for which satisfactory metabolic half-life estimates were obtained.** List was sorted according to half-life estimate value (color coded). Only proteins for which SILAC data was obtained for all 4 time points, and for which fits to single exponentials were acceptable are included in this list. As the data was pooled from multiple experiments data consolidation was necessary. “GI” signifies the protein ID used for such consolidation. “Protein Group (MaxQuant)” signifies the protein groups generated by MaxQuant for MS/MS based protein identifications. “Fraction of heavy AA” is the fractional incorporation of heavy AA at a particular time point (H/(H+L)). “τ” is the time constant of a single exponential function fit to heavy AA incorporation (in days). “t_½_” is the estimated half-life (in days) derived from τ. “SSE” is the sum of square errors for the fits to single exponentials. Maximal accepted SSE value was 0.1.
(XLSX)
