# Supplementary figures and images for: Metabolic Turnover of Synaptic Proteins: Kinetics, Interdependencies and Implications for Synaptic Maintenance
Source: PLoS One. 2013 May 2;8(5):e63191. doi: 10.1371/journal.pone.0063191 (PMC3642143; doi:10.1371/journal.pone.0063191)

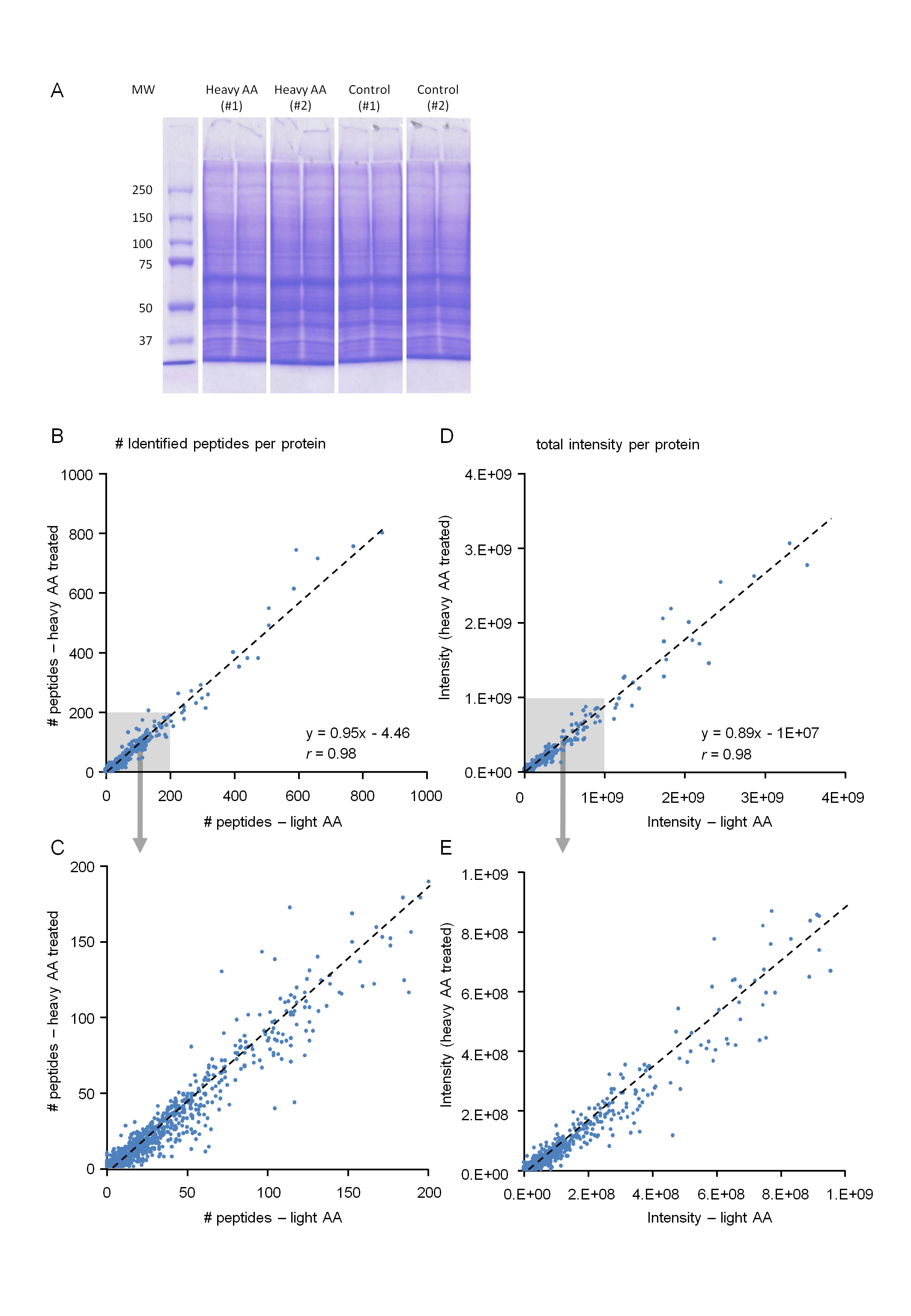

Supplement: Figure S1 — Comparison of MS profiles of labeled and unlabeled samples. Cortical neurons were either grown in the presence of 6× heavy AAs for 3 days (starting at day 14 in vitro as described in main text and in Materials and Methods) or maintained in standard growth media for the same period (control). Lysates of these preparations were then subjected to MS analysis as described in Fig. 1A. Data are from two duplicates, each subjected to separate MS analysis. A) Gel used to separate proteins according to molecular weight (stained with Coomassie Blue; gel image cropped to remove empty lanes). Two lanes were run for each sample to increase protein amounts. Gels were then sliced as in Fig. 1, proteins in each slice were digested, and resulting peptides from each slice and each duplicate were submitted separately to MS analysis. B,C) Number of peptides identified for each protein. Region in gray box is enlarged in bottom panel. Lines represent linear regressions. Data is the sum of peptide numbers identified for each protein in each duplicate. D,E) Total intensities of peptides identified for each protein. Region in gray box is enlarged in bottom panel. Lines represent linear regressions. Data is the average of intensities measured in duplicates. An excellent correlation was observed between MS profiles of heavy AA treated and control preparations. The deviations of the slopes from 1.0 are well within the range observed when comparing two samples. For example, slopes were 1.09 and 1.00 (peptides and intensities, respectively) for control-control duplicate comparisons and 1.15 and 1.34 for heavy AA- heavy AA duplicate comparisons. These differences probably reflect slight differences between the amounts of proteins loaded on the polyacrylamide gels. (TIF) [file pone.0063191.s001.tif]

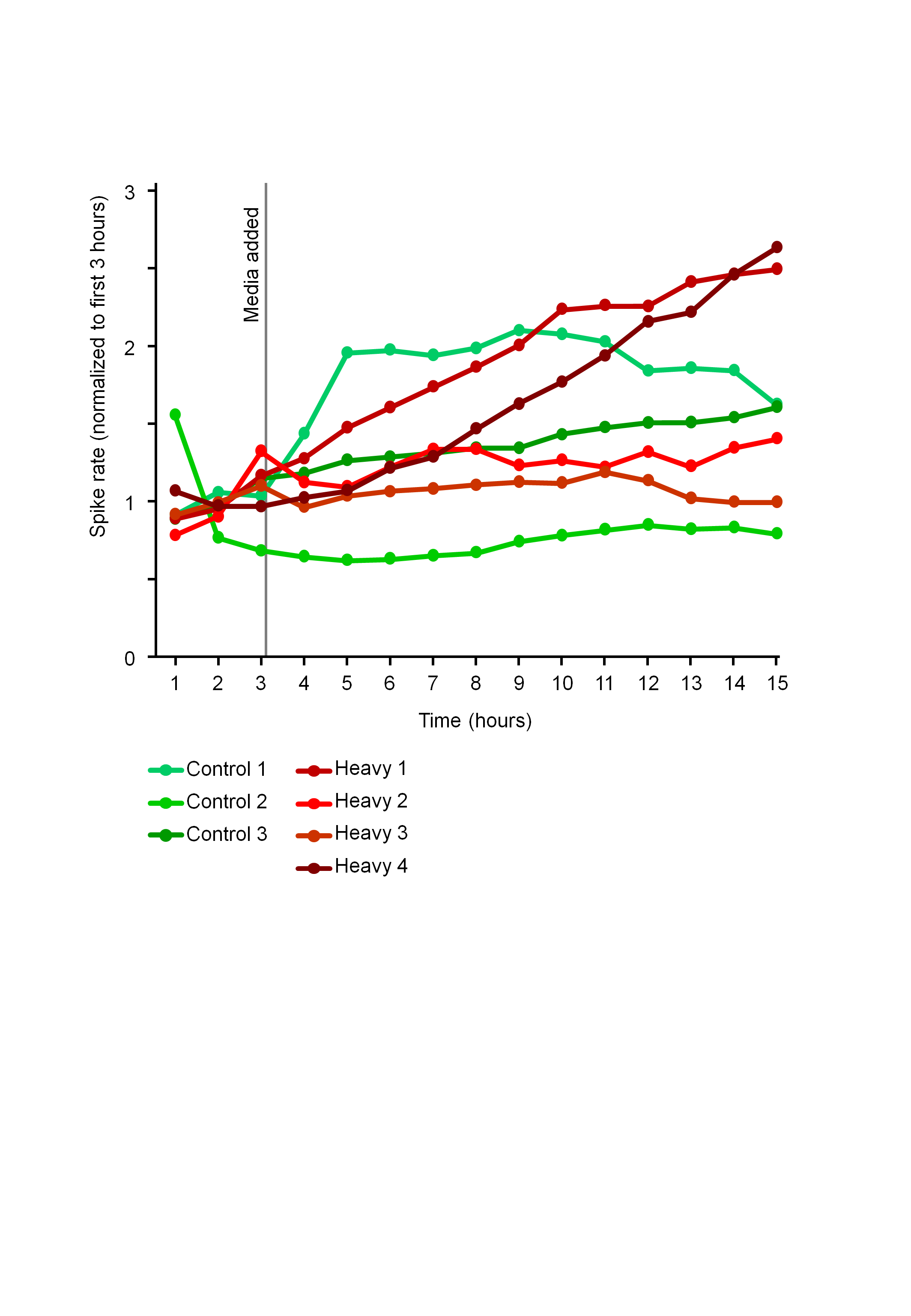

Supplement: Figure S2 — Effects of an excess of heavy AA on network activity. Cortical neurons were grown on multielectrode (MEA) substrates for two weeks in an identical fashion to preparations used for SILAC experiments. The preparations were then mounted on an MEA amplifier as described previously [33]. Spontaneous activity was measured for 3 hours after which heavy AA (Heavy) or standard growth media (Control) were added to the MEA dish in a fashion identical to that performed in SILAC experiments. Recordings were then continued for another 12 hours. Action potentials (spikes) measured from all 60 electrodes were accumulated at 1 min intervals, the resulting spike rates were averaged over one hour time windows and normalized to mean spike rates during first 3 hours. A large variability was observed between networks, but the addition of heavy AA acids did not seem to have any particular effects. (TIF) [file pone.0063191.s002.tif]

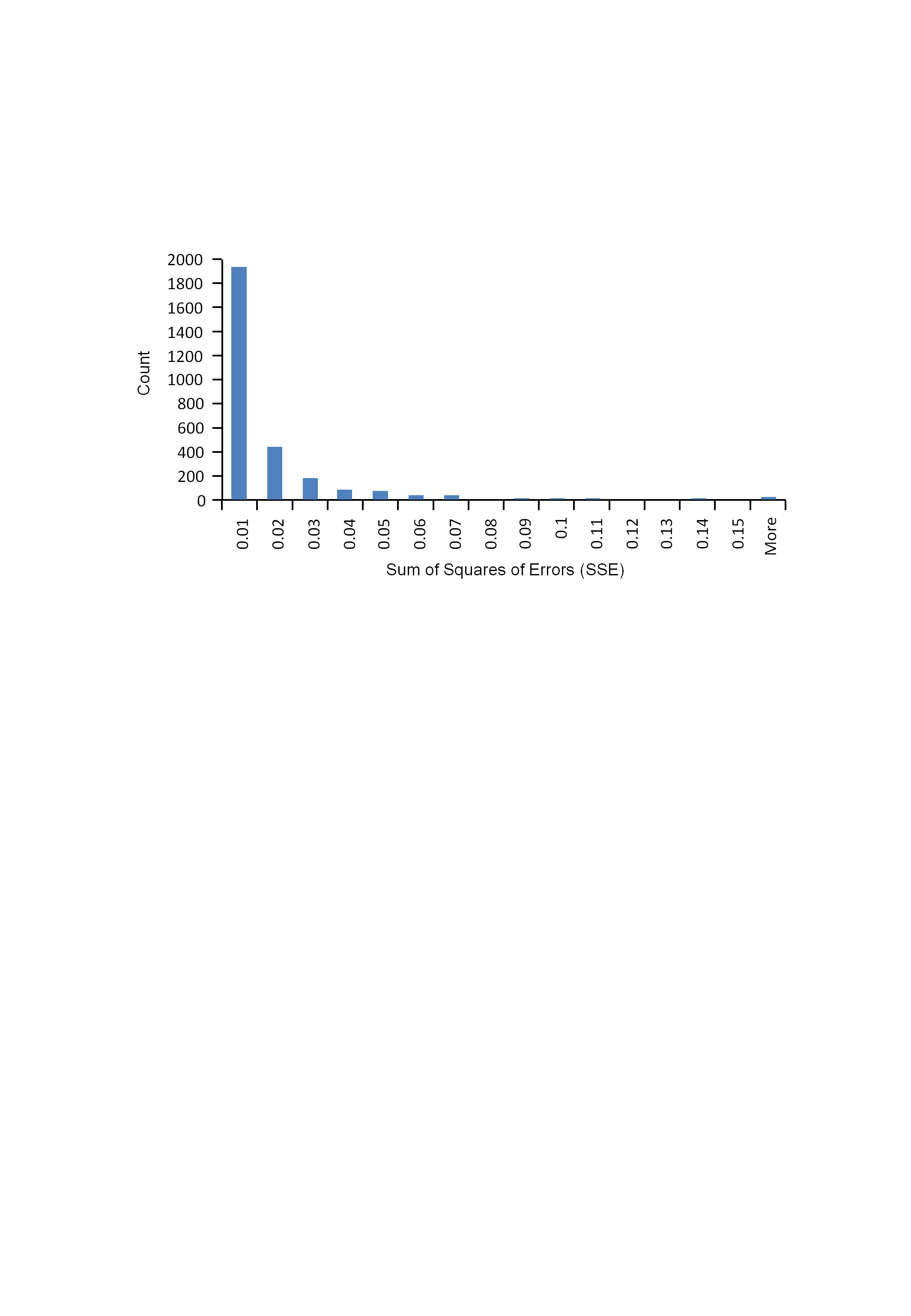

Supplement: Figure S3 — Distribution of sum of square errors (SSE) for fits to single exponentials. SSE values for all identified proteins for which data was obtained for all four time points (2,859 proteins). The lower the SSE value, the better the fit. Note that the fit for the vast majority of proteins was excellent (SSE <0.02) and only a very small number of proteins (∼2%) exhibited unacceptable fits (SSE >0.1). (TIF) [file pone.0063191.s003.tif]

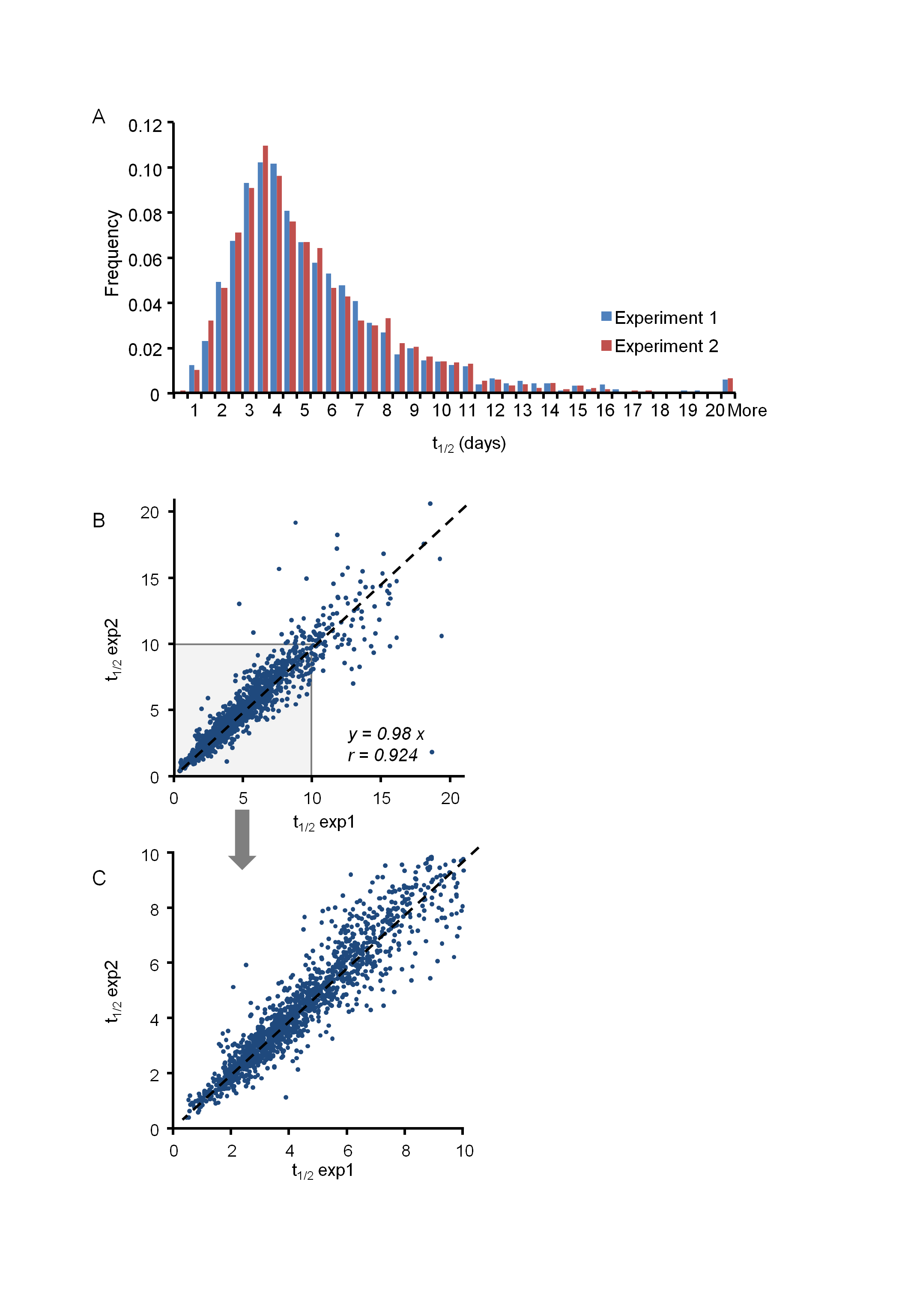

Supplement: Figure S4 — Repeatability of half-life estimations. A) Distributions of half-life estimates obtained separately in two experiments carried out two weeks apart. Only proteins for which data from all 4 time points was obtained in both experiments were included (1,622 proteins). B) Comparison of half-life estimates for individual proteins (1,608). Proteins with half-life estimates exceeding 20 days were excluded. C) Enlargement of region enclosed in gray box in B. (TIF) [file pone.0063191.s004.tif]

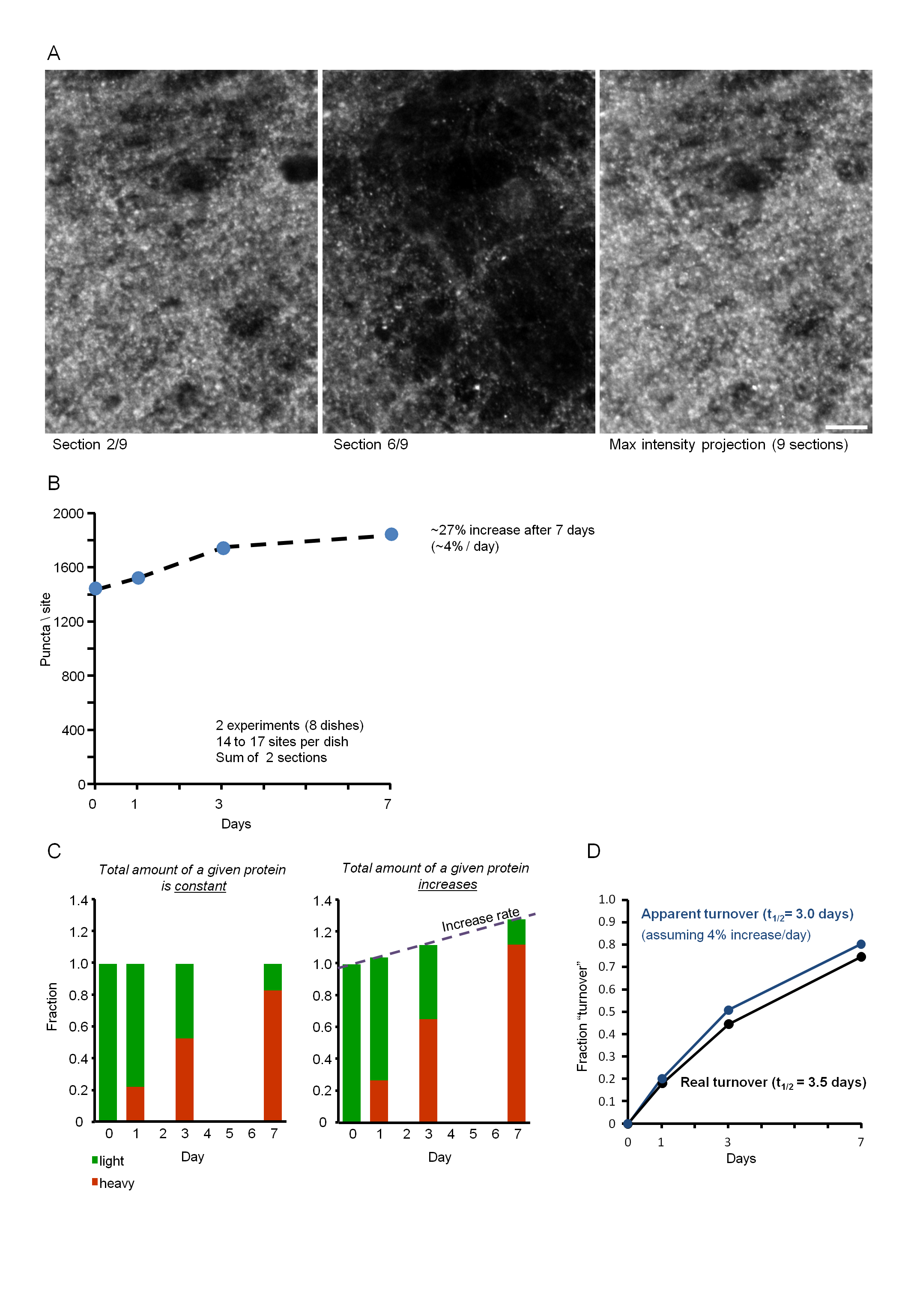

Supplement: Figure S5 — Quantification of synaptic densities at the four time points of the SILAC experiments. Cortical neurons were grown on glass bottom substrates for two weeks in an identical fashion to preparations used for SILAC experiments. The neurons were then fixed after an additional 0, 1, 3 or 7 days and stained against the PSD molecule PSD-95. Nine Z sections were then collected at 0.8 µm intervals at 14 to 17 fields of view in each dish. Synapses were counted programmatically as shown in Fig. 5 in sections #2 or #3 and #6 or #7 and counts were summed, and expressed as average count/field of view for all fields of view in two separate experiments. A) Two representative sections and the maximal intensity projection of all 9 sections. Images were taken at 17 days in vitro (i.e., two weeks +3 days in culture). Bar, 10 µm. B) Changes in synaptic density over the one week period. A mean growth of ∼4%/day in synaptic density was measured. C) Illustration of the effect that increases in total amounts of a given protein will have on half-life estimates. Note that the relative fraction of preexisting protein will seem to decrease (right panel) even though it does not change relative to the left panel. D) The anticipated effect of a 4% growth/day on the half-life estimate for a protein whose “real” half-life is 3.5 days. As shown here, this will lead to an apparent half-life of ∼3.0 days, that is, an overestimation of the turnover rates for this protein. (TIF) [file pone.0063191.s005.tif]

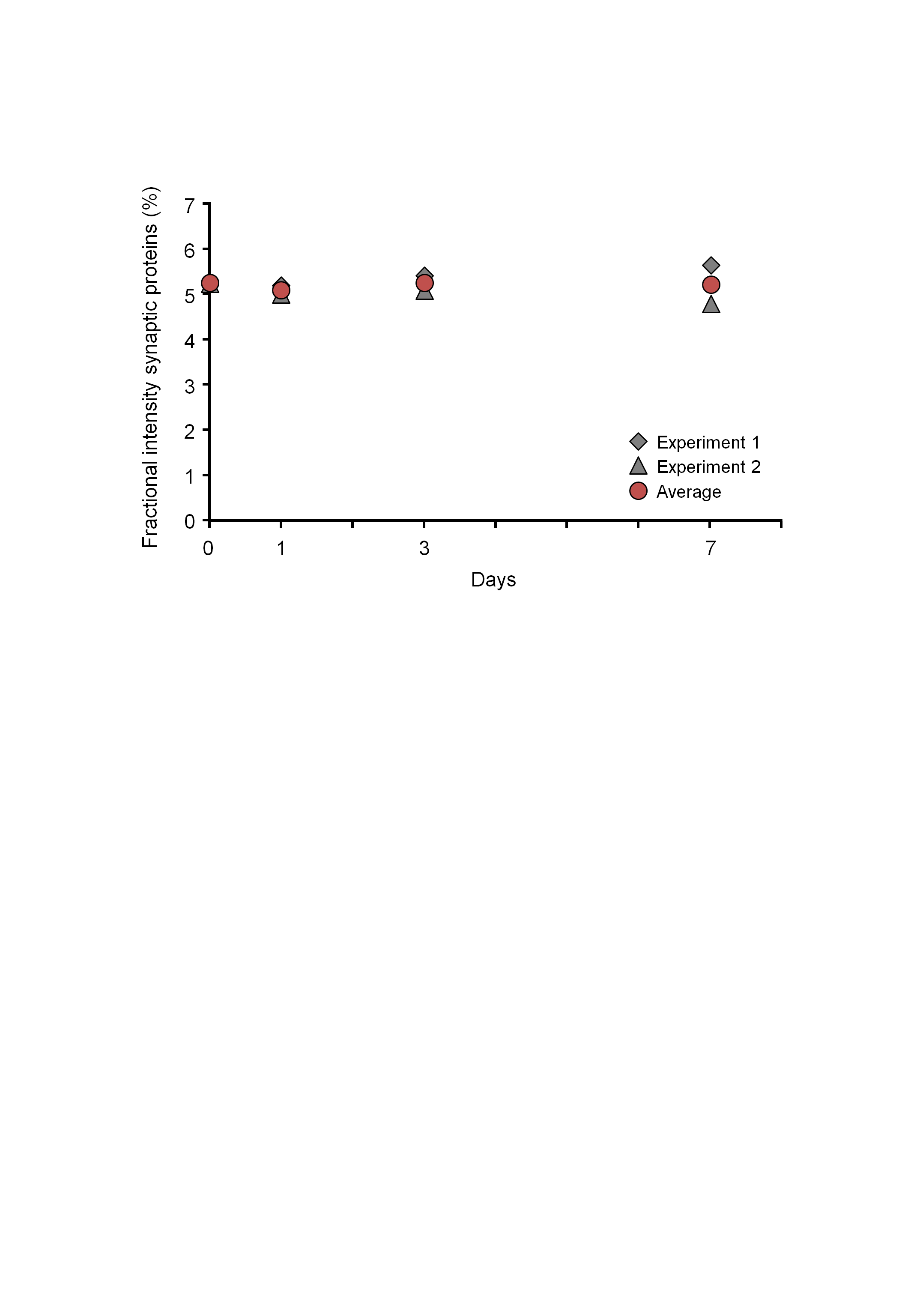

Supplement: Figure S6 — Fractional intensity of synaptic proteins in SILAC experiments. The intensity measured for all peptides (H+L) for all synaptic proteins, divided by the intensities measured for all identified proteins. As equal amounts of proteins were loaded on the separation gels, these data indicate that the fraction of synaptic proteins in the protein mixture barely changed over the 7-day experimental period, arguing against a large increase in synaptic numbers during this period. (TIF) [file pone.0063191.s006.tif]

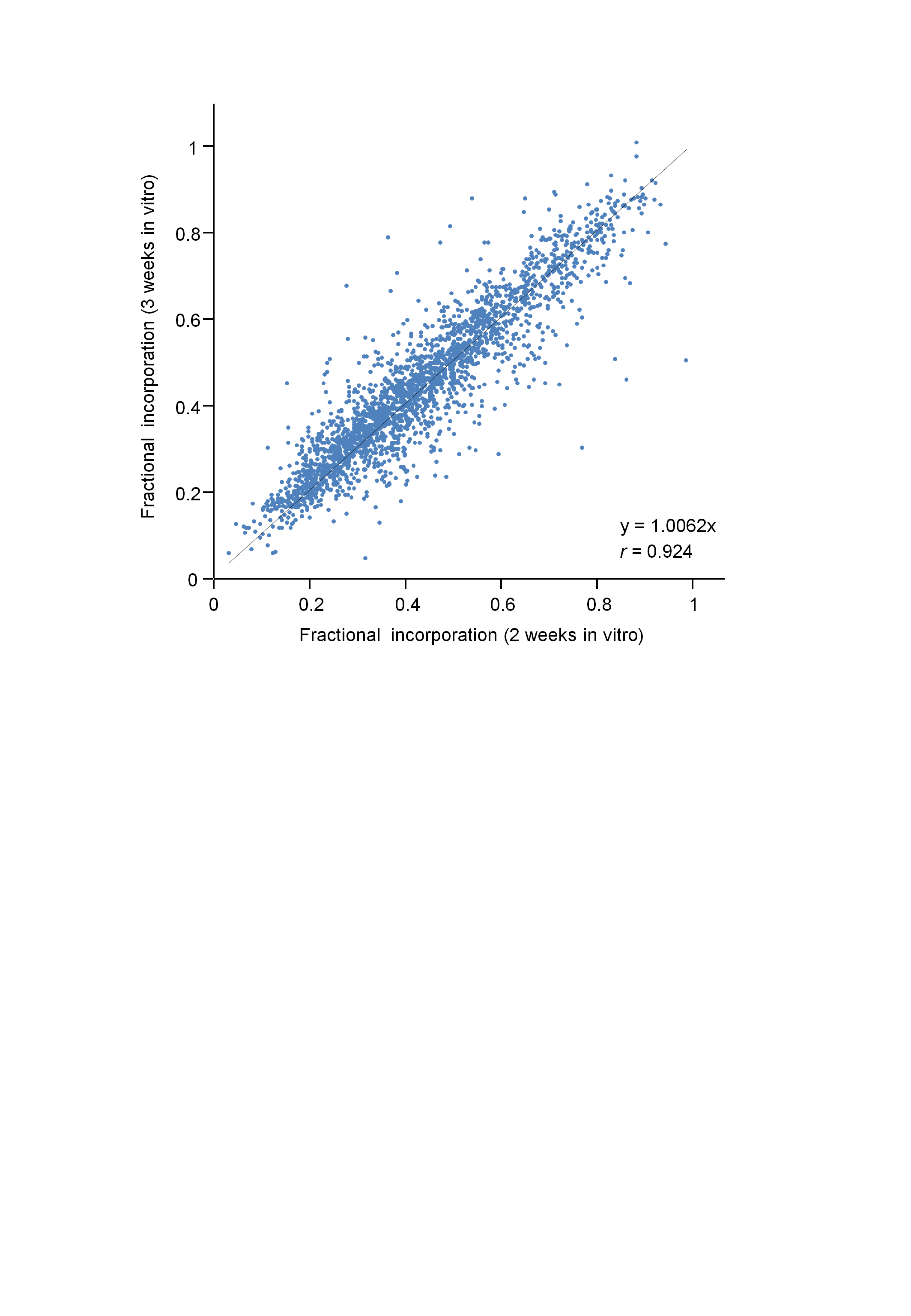

Supplement: Figure S7 — Comparison of fractional incorporation ratios for neurons maintained in culture for 2 weeks and 3 weeks. Cortical neurons were maintained in culture for either 2 weeks or 3 weeks and then exposed to heavy AA for 3 days as described in main text and in Materials and Methods. Lysates of these preparations were then subjected to MS analysis as described in Fig. 1A. The fractional incorporation values were then compared for each protein identified in both data sets (2,460 proteins). Note the good correlation between the two data sets and the fact that the slope is ∼1.0. (TIF) [file pone.0063191.s007.tif]

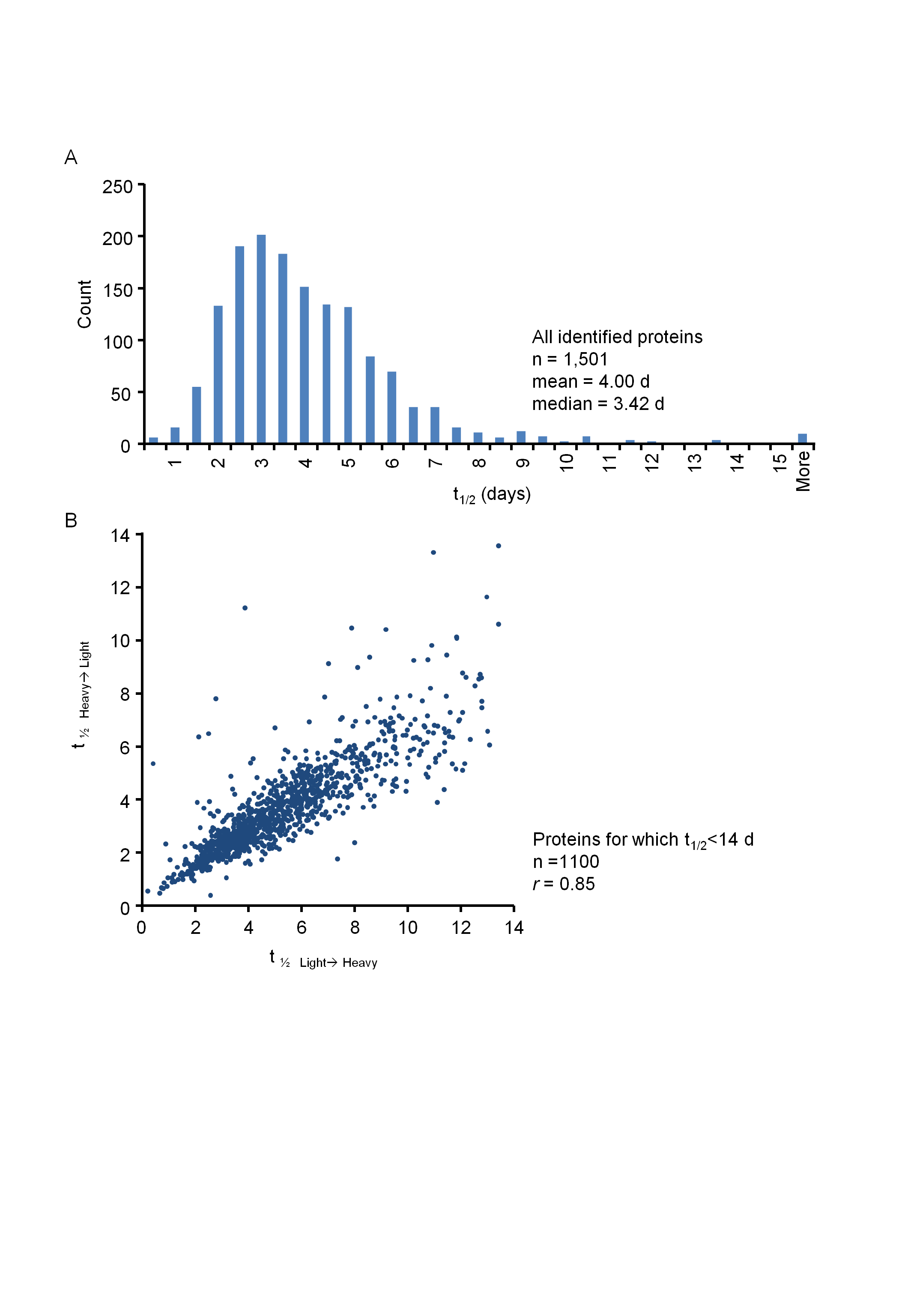

Supplement: Figure S8 — Distributions of metabolic half-life estimates obtained by “conventional” dynamic SILAC. Neurons were grown for 2 weeks in lysine and arginine-free MEM to which Lys8 and Arg10 were added at nominal concentrations. After two weeks, cells were washed and placed in conditioned media from “sister” preparations (see Materials and Methods for details). 0, 1, 3, and 7 days later cells were lysed and processed as described above and half-life estimates were obtained from H/L ratios at 4 time points. A) Distribution of half-life estimates for all proteins for which H/L ratios were obtained at all 4 time points. Only proteins whose fits to single exponentials were satisfactory (SSE <0.1) were included. B) Comparison of half-life estimates for individual proteins for which data was obtained in both forms of dynamic SILAC experiments (Light→Heavy = data of Figs. 1–3; Heavy → Light = data of panel A). (TIF) [file pone.0063191.s008.tif]

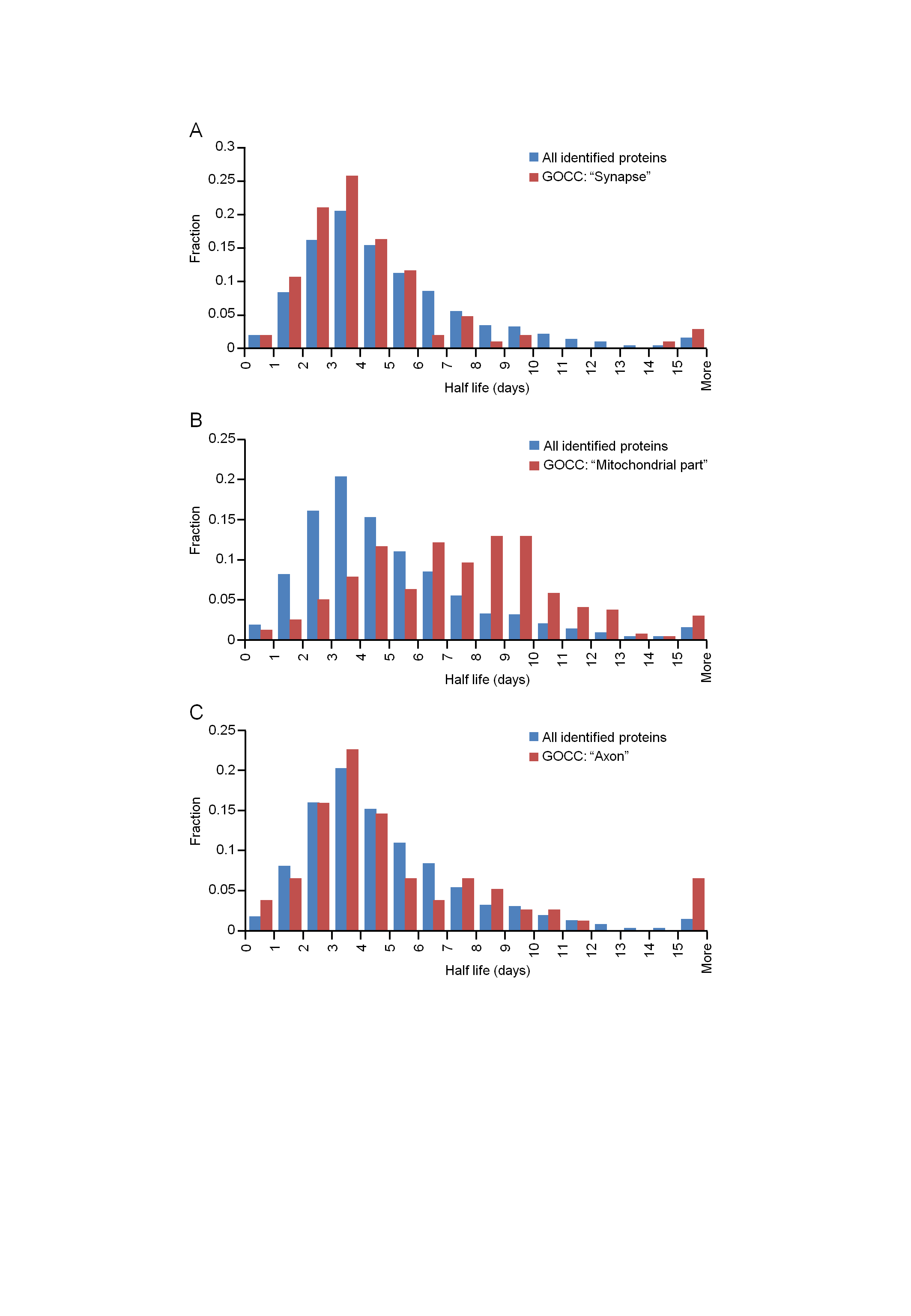

Supplement: Figure S9 — Distributions of metabolic half-life estimates of proteins selected according to particular GO annotations. Subsets of proteins were selected according to specific GO annotations and distributions of their metabolic half-life estimates were compared to those of the entire population (2,804 proteins). A) Cell compartment: “Synapse” (105 proteins). B) Cell compartment: “Mitochondrial part” (240 proteins); C) Cell compartment: “Axon” (75 proteins). (TIF) [file pone.0063191.s009.tif]

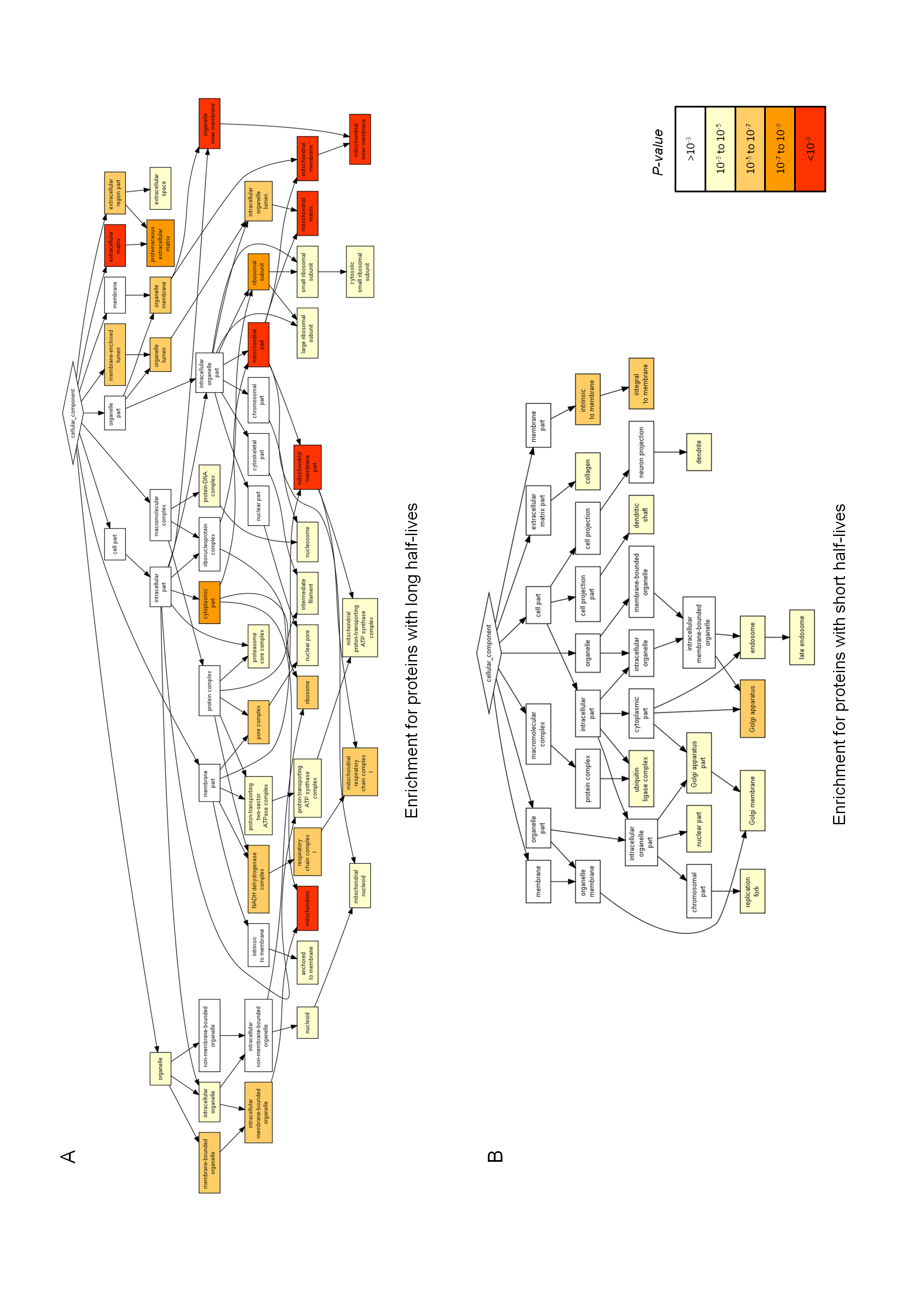

Supplement: Figure S10 — Enrichment analysis of proteins longest and shortest metabolic half-life estimates. Lists of all neuronal proteins for which satisfactory half-life estimates were obtained were sorted according to their half-life estimates and subjected to GO based enrichment analysis (according to cellular component) using the public domain tool “GORILLA” (Gene Ontology enRIchment anaLysis and visuaLizAtion tool; [36]; http://cbl-gorilla.cs.technion.ac.il/). Note that enrichment analysis performed by this tool is based only on rank order, not absolute values. A) Enrichment analysis of proteins with longest metabolic half-life estimates. B) Enrichment analysis of proteins with shortest metabolic half-life estimates. The statistical significance of enrichment scores is color coded according to the index on the right hand side. (TIF) [file pone.0063191.s010.tif]
